# Quantifying exosome secretion from single cells reveals a modulatory role for GPCR signaling

**DOI:** 10.1083/jcb.201703206

**Published:** 2018-03-05

**Authors:** Frederik Johannes Verweij, Maarten P. Bebelman, Connie R. Jimenez, Juan J. Garcia-Vallejo, Hans Janssen, Jacques Neefjes, Jaco C. Knol, Richard de Goeij-de Haas, Sander R. Piersma, S. Rubina Baglio, Matthijs Verhage, Jaap M. Middeldorp, Anoek Zomer, Jacco van Rheenen, Marc G. Coppolino, Ilse Hurbain, Graça Raposo, Martine J. Smit, Ruud F.G. Toonen, Guillaume van Niel, D. Michiel Pegtel

**Affiliations:** 1 Department of Pathology, Cancer Center Amsterdam, VU University Medical Center, Amsterdam, Netherlands; 2 Department of Medical Oncology, Cancer Center Amsterdam, VU University Medical Center, Amsterdam, Netherlands; 3 Department of Molecular Cell Biology and Immunology, VU University Medical Center, Amsterdam, Netherlands; 4 Department of Clinical Genetics, VU University Medical Center, Amsterdam, Netherlands; 5 Division of Medicinal Chemistry, Amsterdam Institute for Molecules Medicines and Systems, VU University Amsterdam, Amsterdam, Netherlands; 6 Department of Functional Genomics, Center for Neurogenomics and Cognitive Research, VU University Amsterdam, Amsterdam, Netherlands; 7 Division of Cell Biology, Netherlands Cancer Institute, Amsterdam, Netherlands; 8 Department of Chemical Immunology, Leiden University Medical Center, Leiden, Netherlands; 9 Cancer Genomics Netherlands–Hubrecht Institute–Koninklijke Nederlandse Akademie van Wetenschappen, Utrecht, Netherlands; 10 University Medical Centre Utrecht, Utrecht, Netherlands; 11 Department of Molecular and Cellular Biology, University of Guelph, Guelph, Canada; 12 Institut Curie, Paris Sciences et Lettres Research University, Centre National de la Recherché Scientifique, UMR 144, Paris, France; 13 Centre National de la Recherché Scientifique, UMR 144, Paris, France; 14 Cell and Tissue Imaging Core Facility PICT-IBiSA, Institut Curie, Paris, France

## Abstract

Exosomes are small endosome-derived extracellular vesicles implicated in cell–cell communication and are secreted by living cells when multivesicular bodies (MVBs) fuse with the plasma membrane (PM). Current techniques to study exosome physiology are based on isolation procedures after secretion, precluding direct and dynamic insight into the mechanics of exosome biogenesis and the regulation of their release. In this study, we propose real-time visualization of MVB–PM fusion to overcome these limitations. We designed tetraspanin-based pH-sensitive optical reporters that detect MVB–PM fusion using live total internal reflection fluorescence and dynamic correlative light–electron microscopy. Quantitative analysis demonstrates that MVB–PM fusion frequency is reduced by depleting the target membrane SNAREs SNAP23 and syntaxin-4 but also can be induced in single cells by stimulation of the histamine H1 receptor (H1HR). Interestingly, activation of H1R1 in HeLa cells increases Ser110 phosphorylation of SNAP23, promoting MVB–PM fusion and the release of CD63-enriched exosomes. Using this single-cell resolution approach, we highlight the modulatory dynamics of MVB exocytosis that will help to increase our understanding of exosome physiology and identify druggable targets in exosome-associated pathologies.

## Abstract (precis)

All mammalian cells release small endosome-derived exosomes that function in intercellular communication, but the secretion process is poorly understood. Verweij et al. developed a live-imaging approach and demonstrate that external cues can trigger exosome release from a subpopulation of multivesicular bodies by phosphorylating the target membrane SNARE SNAP23 at serine residue 110.

## Introduction

Extracellular vesicles (EVs) have a growing inventory of biological functions, and their mechanisms of biogenesis are currently intensively studied (Tkach and Théry, 2016). Although EVs are generally studied indiscriminately as a collection of subtypes, much attention has been drawn to intracellularly generated exosomes and EVs that bud from the plasma membrane (PM), typically designated as microvesicles (MVs). It has proven difficult to attribute distinct physiological functions to either exosomes or MVs because both EV subtypes share many features in their biogenesis and have many cargo molecules in common. Exosomes originate as intraluminal vesicles (ILVs) within late endosomal compartments called multivesicular bodies (MVBs). Exosomes are formed by inward budding of the limiting membrane into the lumen, which can be ESCRT-dependent or -independent (van Niel et al., 2011; Colombo et al., 2014). When MVBs fuse with the PM, exosomes are released, although depending on the cell type, a proportion might remain attached to the cell surface (Edgar et al., 2016). Once released from producer cells, exosomes may operate in a variety of fundamental biological processes including development, stemness maintenance, and immune responses as well as in pathologies (Tkach and Théry, 2016). Despite a broad knowledge of the biomolecules packaged within EVs, the mechanisms that control MVB–PM fusion, the step preceding exosome release, remain poorly understood, hampering physiological studies into the role of exosomes in vivo.

Whereas neurotransmitter release is widely studied by live imaging of single neuronal cells (Mohrmann et al., 2010), exosome release is typically studied biochemically upon collection of cell culture supernatant over extended time periods (24–72 h). This approach has a crucial drawback in that the dynamic aspects of exosome release and potential heterogeneity in vesicle production are ignored. Moreover, long-term stimulation in culture likely does not capture subtle signaling events that control membrane fusion required for exosome release. Indeed, thus far, a direct demonstration that inducible signaling pathways function as triggers for MVB–PM fusion has been lacking.

Being interested in capturing the dynamics of exosome secretion, we reasoned that direct visualization and quantification of MVB–PM fusion could yield novel mechanistic insights. We designed pH-sensitive tetraspanin (TSPAN) reporters that were expressed in HeLa cells for live- and correlative light–EM (CLEM) that captured MVB–PM fusion in supraoptical EM resolution. We determined that MVB–PM fusion is controlled by SNARE molecules and becomes more frequent upon GPCR signaling from the histamine H1 receptor (H1HR) via phosphorylation of serine residue 110 of the t-SNARE SNAP23. Importantly, the stimulation of exosome release studied in this paper appears distinct from classical Ca^2+^-triggered activation of SNARE fusion machineries that mediate neurotransmitter release (Hay, 2007), neutrophil secretion (Karim et al., 2013), or exocytosis from secretory lysosomes (Rodríguez et al., 1997). Collectively, our work suggests that a significant proportion of fusion-competent MVBs are responsive to external cues for externalization of exosomes, shedding new light on the physiological role of exosomes as cell–cell communication devices in vivo.

## Results

### Development of a pH-sensitive reporter for MVB–PM fusion

**Figure 1.**
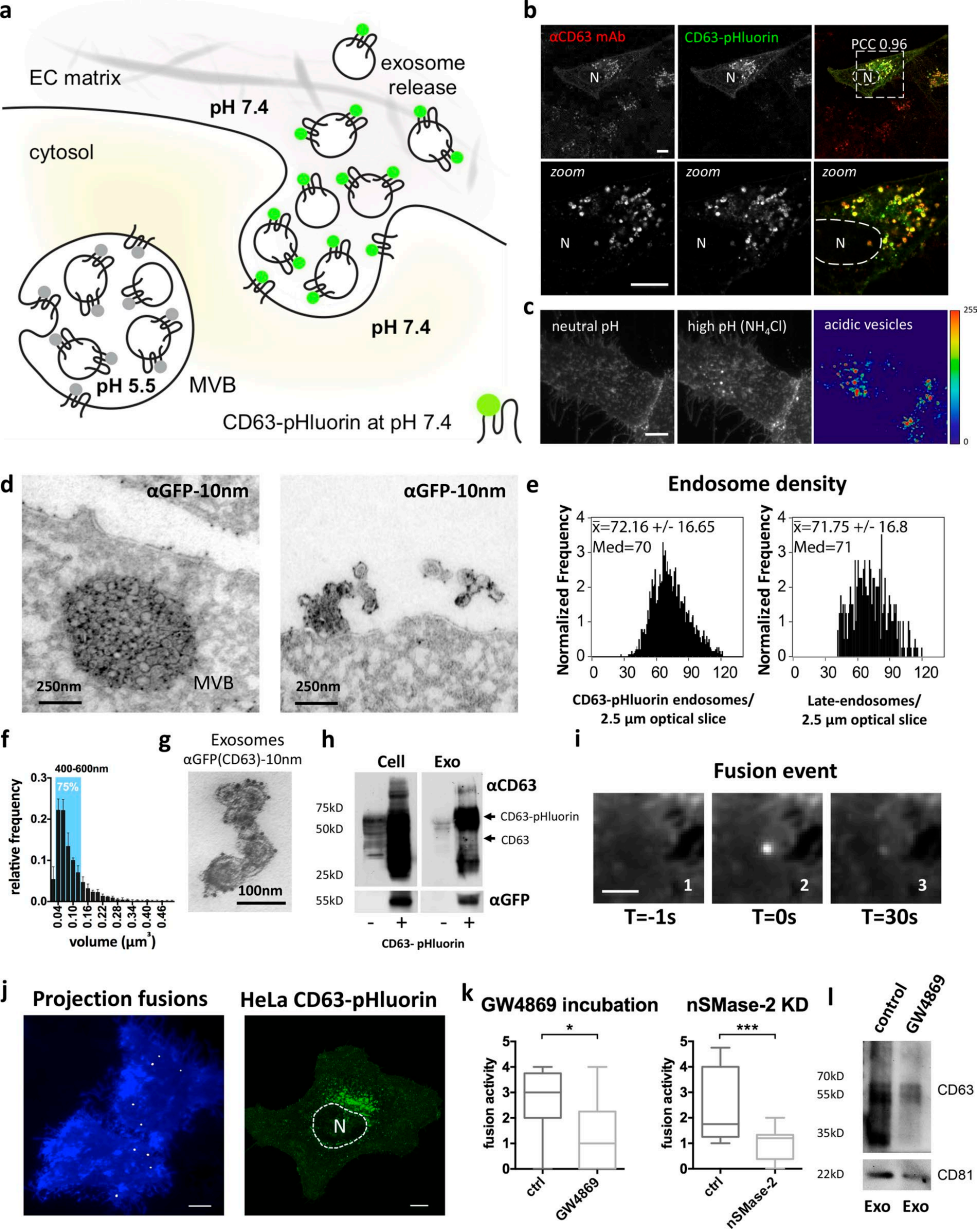
**CD63-pHluorin is sorted into acidic MVBs and released via exosomes. (a)** Proposed model for the visualization of MVB–PM fusion: a pH-sensitive optical reporter (CD63-pHluorin) is quenched when facing the acidic lumen of the MVB. Upon fusion, low luminal pH is immediately neutralized, resulting in a sudden increase in fluorescent intensity. EC, extracellular. **(b)** Immunofluorescent colabeling of total CD63 (red) and CD63-pHluorin (green) in HeLa cells. PCC, Pearson’s correlation coefficient. **(c)** TIRF images of a CD63-pHluorin–expressing HeLa cell at normal and elevated intracellular pH (NH_4_Cl superfusion). On the right, a heat map revealing acidic vesicles close to the PM was obtained by subtracting the fluorescent intensity values of the normal pH from the high-pH condition. **(d)** EM images of an MVB close to the PM (left) and EVs aligning the PM (right) labeled with gold particles directed to GFP (10 nm) in CD63-pHluorin–expressing HeLa cells. **(e)** Imaging flow cytometry of the number of late endosomes per cell in a 2.5-µm optical section in CD63-pHluorin–expressing cells (left) or immunostaining against LAMP1 in nontransfected cells (right; *n* > 2,000 cells). **(f)** Volume distribution of endosomes based on analysis of whole-cell confocal scans (error bars represent SD; *n* = 3). The blue area accounts for 75% of the total number of endosomes and covers the 400–600-nm-diameter range. **(g)** Immunogold labeling on purified exosomes with gold particles (10 nm) coupled to anti-GFP antibody. **(h)** Western blotting analysis on untransfected (−) and CD63-pHluorin–transfected (+) cells and purified exosomes for total CD63 and GFP. **(i)** Example of a localized sudden increase in fluorescence at the PM before the event (1), during the event (2), and right before disappearance of the signal (3). **(j)** Left: total projection of fusion events (bright spots) over a time course of 3 min onto two cells (blue). Right: representative example of CD63-pHluorin–expressing HeLa cell. N, nucleus. Bars: (b, c, and j) 10 µm; (i) 2.5 µm. **(k)** Effect of incubation with GW4896 (5 µM; *n* ≥ 8 cells per condition) and nSMase-2 knockdown (*n* ≥ 22 cells per condition) on fusion activity in HeLa cells. *, P < 0.05; ***, P < 0.001 using Student’s two-tailed two-sample *t* test. Whiskers in the box plots represent 1.5 times the interquartile distance or the highest or lowest point, whichever is shorter. **(l)** Western blotting analysis on purified exosomes from GW4896- and control-treated HeLa cells for CD63 and CD81.

We designed TSPAN-based optical reporters with a pH-sensitive GFP (pHluorin) cloned in the first extracellular loop (ECL1) of the molecule to visualize endosome fusion with the PM (for a schematic, see Fig. 1 a). We first verified proper trafficking and localization of the CD63-pHluorin reporter when expressed in HeLa cells with light microscopy and EM analysis. We observed presence of the reporter in the limiting membrane and enrichment in ILVs of acidic MVBs and on vesicles tethered to the cell surface as recently reported (Fig. 1, b–d; and Fig. S1 a, top; Edgar et al., 2016). Using imaging flow cytometry, we detected ∼70 CD63-pHluorin–rich compartments per 2.5-µm slice, which can be extrapolated to 200 per cell (Fig. 1 e), in line with the mean number of endogenous late endosomes (Schauer et al., 2010). Size/volume calculations based on whole-cell scans suggest that the majority (75%) of intracellular CD63-pHluorin–rich vesicles are in the MVB size range of 400–600 nm diameter as confirmed by EM (Figs. 1 f and S1 b). Purification of EVs in the supernatant by differential ultracentrifugation (Fig. S1 c; Verweij et al., 2013) confirmed the release of CD63-pHluorin–rich exosomes by immuno-EM (Figs. 1 g and S1 a, bottom) and Western blotting analysis (Fig. 1 h). (Of note, recognition of this construct by anti-CD63 antibody suggested no obvious changes in the conformation of the protein.) Finally, comparison between CD81 levels in exosomes isolated from CD63-pHluorin–transfected and –nontransfected cells suggested that there was no significant increase in the total number of released exosomes as a result of expression of the construct (Fig. S1 d). These data indicate that expression of this reporter does not affect either the trafficking of CD63 or the number and the morphology of MVBs.

To investigate the sensor potential of CD63-pHluorin in living HeLa cells, we performed total internal reflection fluorescence (TIRF) microscopy that was optimized for high-resolution detection of fluorescent signals at or near the PM (Miesenböck et al., 1998). In cells expressing moderate levels of CD63-pHluorin, we could detect multiple localized increases in fluorescence signal at the PM over time (Fig. 1, i and j, left), suggesting fusion of CD63-pHluorin–positive acidic vesicles with the PM. To assess whether the fusion spots may report exosome release, we inhibited the enzyme-neutral sphingomyelinase 2 (nSMase-2) with GW4869 and RNA silencing of nSMase-2, known to decrease exosome release (Trajkovic et al., 2008). We found that both GW4869 treatment and nSMase-2 knockdown significantly decreased the number of fusion events (Fig. 1 k). Importantly, we could confirm that the diminished fusion activity upon GW4869 treatment resulted in a significant decrease in secreted exosomes as determined by Western blotting for CD63 and CD81 (Fig. 1 l).

Thus, CD63-pHluorin appears to be a suitable optical reporter of MVB–PM fusion events, and as a corollary, exosome release. CD63-pHluorin was recently used as a tool to mark externalization of CD63-positive structures at sites of adhesion assembly (Sung et al., 2015). In a time-lapse recording of 3 min, however, we observed multiple fusion events scattered across the PM (Fig. 1 j, left; and Video 1). Moreover, even though the majority of CD63 endosomes was localized around the nucleus (Fig. 1 j, right), we did not observe preferential sites for MVB–PM fusion. A similar phenotype of fusion events was observed for SiHa cells (Video 2), primary human umbilical vein endothelial cells (HUVECs; Fig. S1 e and Video 3), and mesenchymal stem cells (MSCs; Video 4), although the frequency of fusion events in these nontransformed cells was generally lower compared with HeLa cells.

Finally, to confirm that fusion events reported by CD63-pHluorin correspond with late endosomes externalizing vesicular cargo, we designed a CD63-pHluorin variant (CD63–C-term–pHluorin) with pHluorin placed at the C terminus. When this reporter is present in the limiting membrane of an intracellular compartment, the pHluorin moiety will keep facing the cytoplasm and its neutral pH during fusion with the PM. Although CD63–C-term–pHluorin that is sorted into ILVs faces the lumen of ILVs, it will be sensitive to pH changes during MVB–PM fusion as phospholipid bilayers are permeable to protons (Raven and Beardall, 1981; Gutknecht, 1987; Paula et al., 1996). Thus, CD63–C-term–pHluorin will only generate a burst of fluorescence at the PM during endosome–PM fusion events if ILVs are exposed to the extracellular neutral pH (Fig. S2, a and b). Western blot analysis of isolated exosomes confirmed the release of CD63–C-term–pHluorin in exosomes along with endogenous CD63 (Fig. S2 c). Coexpression of CD63–C-term–pHluorin with a CD63 construct containing a pH-sensitive red fluorescent version of pHluorin in ECL1 (CD63-pHuji) revealed that the fusion events of the CD63-pHuji reporter coincided with bursts of fluorescence of the CD63–C-term–pHluorin, indicating cargo externalization in the majority (>95%) of CD63–pHluorin fusion events (Fig. S2 b and Video 5). These findings suggest that CD63-pHluorin is an optical reporter for the externalization of exosomes.

### Capture of an MVB–PM fusion profile by dynamic CLEM

**Figure 2.**
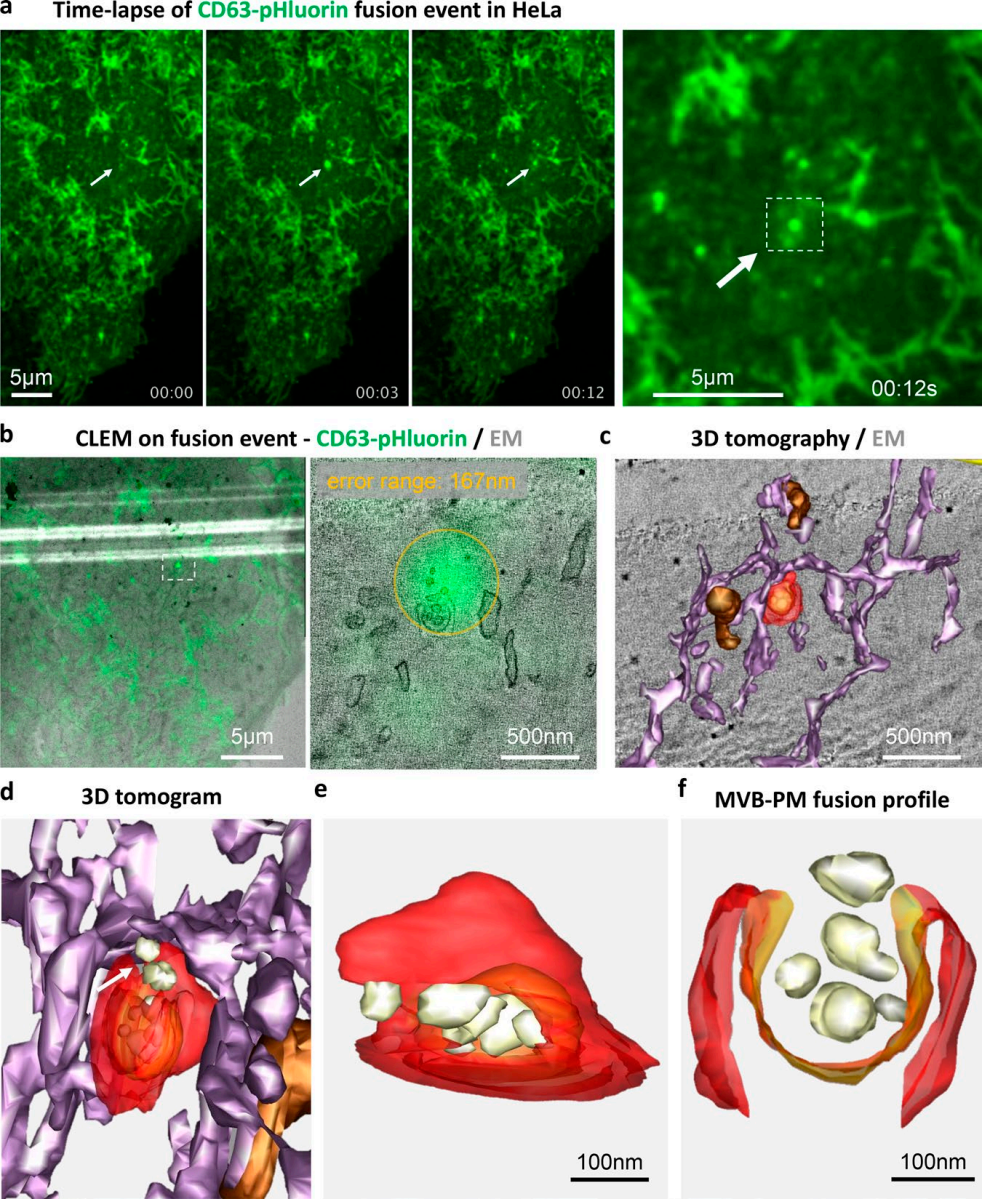
**CD63-pHluorin fusion events are derived from MVBs. (a)** Left three panels: live imaging of fusion events (indicated by white arrows) over a time course of 12 s onto one cell before the event (left), at the start of the event (middle), and right before fixation of the cell (3). Right: inset showing a magnification of the localized sudden increase in fluorescence at the PM (highlighted by a dashed line square) right before fixation. **(b)** Left: correlation of light microscopy signal of a fusion event observed by live imaging with EM pictures of the first section of the cell facing the coverslip (low magnification). Right: correlation of light microscopy signal with the first slice of the electron tomographic reconstruction of the first section of the cell facing the coverslip. The orange circle indicates the error range (167 nm) of the correlation performed by eC-CLEM. **(c)** 3D model of the electron tomographic reconstruction. The ER is depicted in light violet. Dense compartments are depicted in brown. The structure of interest is depicted in red and orange. **(d)** Bottom side view of the 3D model of the compartment of interest in its surroundings. The white arrow indicates the opening of the MVB where ILVs are released. **(e)** 3D model showing the MVB isolated from its environment. ILVs secreted through the opening of the MVB are depicted in white. **(f)** Top view of the secretory profile of the MVB that correlates with the fluorescence burst of the CD63-pHluorin fusion event.

To confirm that the observed bursts in fluorescence at the PM indeed represent fusion of MVBs with the PM followed by externalization of ILVs, we took a dynamic CLEM approach. The procedure is explained in Video 6. In short, live HeLa cells expressing our CD63-pHluorin reporter for MVB–PM fusion were imaged and chemically fixated within seconds after the start of a burst of fluorescence at the PM facing the glass side of the imaging chamber (Fig. 2 a) and then were further processed for EM imaging. The region of interest of the PM facing the coverslip was analyzed by electron tomography. Using recently designed software (Paul-Gilloteaux et al., 2017), we could correlate the burst of fluorescence with the tomogram at an error range of 167 nm (Fig. 2 b). Subsequent 3D reconstruction revealed that the burst of fluorescence corresponded with a fusion event of an MVB with the PM-externalizing ILVs (Fig. 2, c–f). This unique dynamic CLEM analysis of the CD63-pHluorin burst confirmed the nature of the fluorescent spots detected at the PM as fusion events between MVBs and PMs.

### CD63- and CD81-pHluorin have distinct fusion characteristics compared with vesicle-mediated secretion of soluble proteins or PM deposition

**Figure 3.**
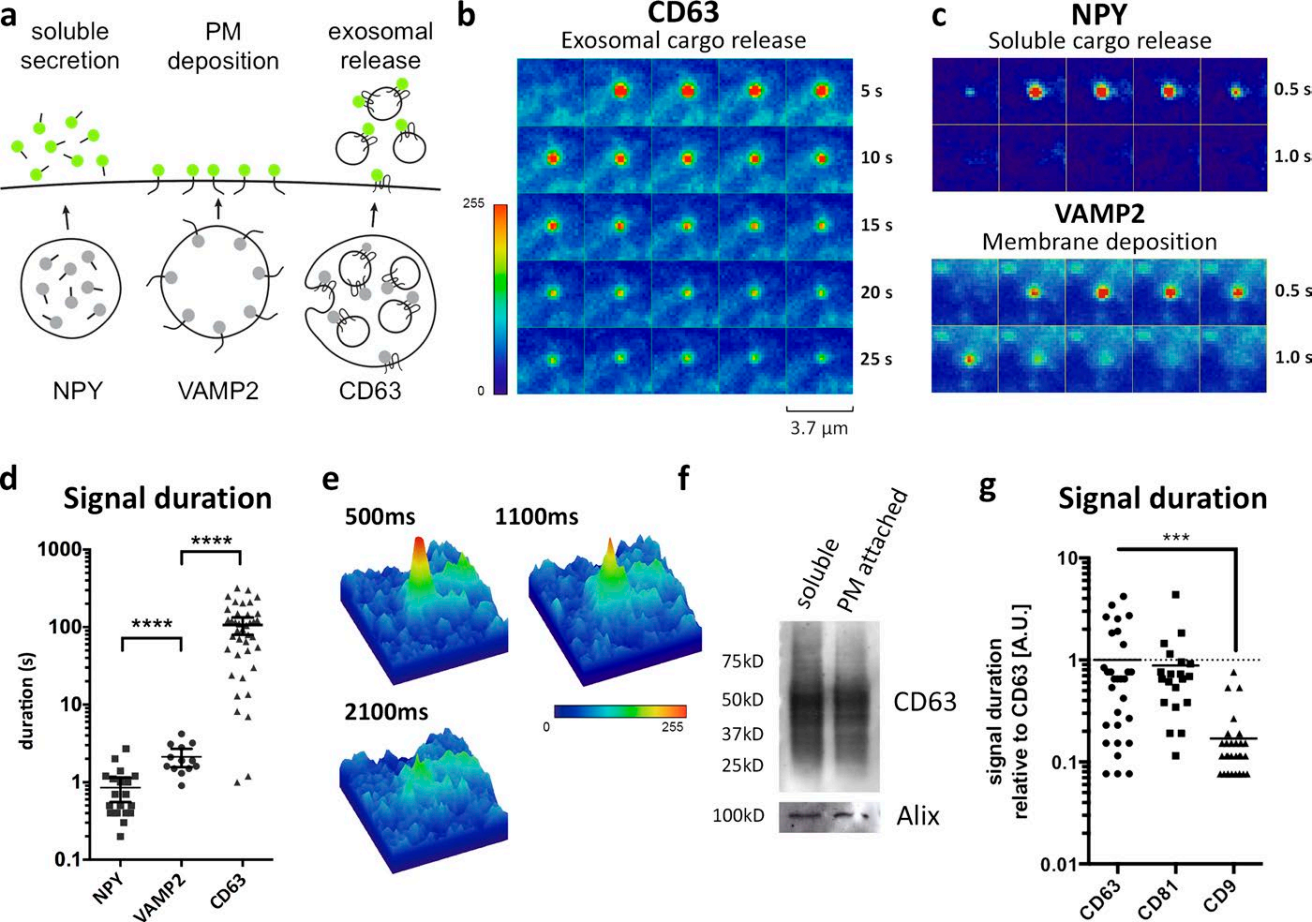
**MVB–PM fusion is distinct from other forms of vesicle-mediated exocytosis. (a)** Schematic model showing the markers used in this study for the different types of cargo delivery of vesicles fusing with the PM. **(b)** Time-lapse imaging (heat maps) of a fusion event of the exosomal protein CD63-pHluorin. **(c)** Time-lapse images of soluble (NPY-pHluorin) and membrane protein (VAMP2-pHluorin) fusion events. **(d)** Fluorescent signal duration of NPY (mean = 0.85 s), VAMP2 (mean = 2.12 s), and CD63 (mean = 106.55 s) fusion events. *n* ≥ 13 events per reporter. **(e)** 3D heat maps of three consecutive CD63-pHluorin fusion event frames. **(f)** Western blot for exosomal markers (CD63 and Alix) on EVs purified from the supernatant (soluble) and EVs attached to the cell surface (PM attached) isolated after short trypsinization of the cells. **(g)** Direct comparison between signal duration of fusion events of CD81- and CD9-pHluorin relative to CD63-pHluorin. *n* ≥ 20 events per reporter. ***, P < 0.001; ****, P < 0.0001 using Student’s two-tailed two-sample *t* test.

To determine whether the fusion characteristics are typical of MVB-associated CD63-pHluorin, we generated CD81-pHluorin and CD9-pHluorin. Like CD63, CD81 is associated with MVBs and enriched in 100-nm exosome-like vesicles, whereas CD9 appears enriched in small EVs that bud from the PM (Kowal et al., 2016). We first investigated post-fusion dynamics of the TSPAN reporters and compared this to soluble protein secretion of neuropeptide Y (NPY) and PM deposition of the integral synaptic vesicle protein VAMP2, also tagged with pHluorin (Fig. 3, a–c; and Videos 1, 7, and 8). CD63-pHluorin bursts showed an extended fluorescence signal compared with the short-lived fluorescence increase associated with the release of the soluble cargo (NPY) or with membrane deposition (VAMP2; Fig. 3, b–e). In line with CD63–C-term–pHluorin–induced bursts and EM and CLEM data, we concluded that CD63-pHluorin bursts unlikely result from the release of cleaved pHluorin or PM deposition of molecules derived from limiting membranes of secretory organelles. Potential explanations of the very long signal duration may be entrapment of fluorescent exosomes between the cells and the plastic support or their immobilization by “sticking” to the PM after MVB–PM fusion (Video 9; Edgar et al., 2016). We tested the latter option by short trypsinization of cells and subsequent purification of cell-associated EVs with differential ultracentrifugation (Fig. S3 a). We could indeed detect EV-associated CD63-pHluorin physically attached to the cell surface (Fig. 3 f). Nevertheless, a knockout of the GPI anchor protein tetherin did not affect the signal duration for CD63 (Fig. S3 b), excluding a tetherin-mediated tethering of exosomes to the PM (Edgar et al., 2016). The prolonged fluorescent signals at the PM from CD63-pHluorin were also observed with CD81-pHluorin but not with the CD9-pHluorin reporter in HeLa cells (Fig. 3 g and Videos 1, 10, and 11). These differences were observed in other cell types as well (Fig. S3 c). We speculate that the different kinetics between TSPAN-pHluorins may reflect secretion on ILVs for CD63 and CD81 and PM deposition for CD9 (Kowal et al., 2016). However, MVBs with a different composition and size may also impact signal duration upon fusion with the PM. In conclusion, we propose that MVB–PM fusion kinetics are distinct from those of transport vesicles that mediate soluble protein release and PM deposition.

### Histamine-mediated GPCR activation triggers MVB–PM fusion

**Figure 4.**
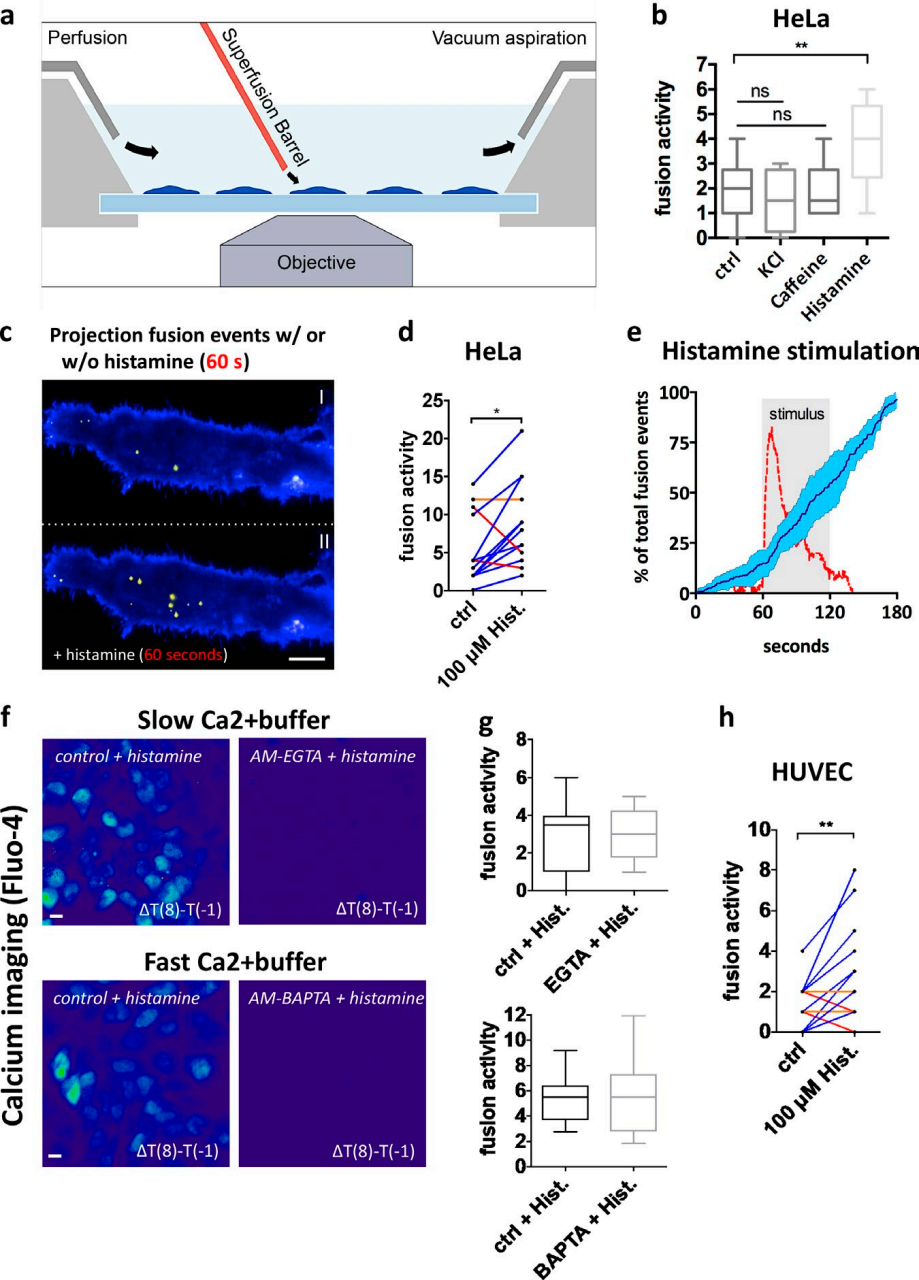
**GPCR activation triggers MVB–PM fusion in single cells in a calcium-independent manner. (a)** Schematic model of imaging setup. **(b)** Fusion activity of HeLa cells stimulated with KCl (70 mM), caffeine (20 mM), or histamine (100 µM). *n* ≥ 8 cells per condition. **(c)** Total projection of fusion events over a 60-s time course onto cells before (top) and after (bottom) stimulation with histamine (100 µM). Pseudocolored as in Fig. 1 j. **(d)** Measurement of individual HeLa cells (*n* = 14) before and during stimulation with histamine (100 µM). **(e)** Mean fusion kinetics of CD63-pHluorin HeLa cells (*n* = 6) showing the distribution of fusion events over time (dark blue line; SD is in light blue) and the calcium levels (red) during histamine stimulation (gray-shaded block). **(f)** Heat maps revealing calcium responses (measured by Fluo-4) upon histamine stimulation obtained by subtracting the fluorescent intensity values before stimulation from those after 8-s stimulation. Cells were nontreated or incubated with a buffer with fast (BAPTA) or slow (EGTA) calcium-binding kinetics. Bars, 10 µm. **(g)** Quantification of fusion activity of histamine-stimulated HeLa cells in the presence of EGTA (top) or BAPTA (bottom) buffers. *n* ≥ 10 cells per condition. **(h)** Measurement of individual HUVEC cells (*n* = 30) before and after stimulation with histamine (100 µM). *, P < 0.05; **, P < 0.01 using Student’s two-tailed two-sample *t* test. All *t* tests were unpaired except for d and h. Whiskers in the box plots (b and g) represent 1.5 times the interquartile distance or the highest or lowest point, whichever is shorter.

Based on ELISA experiments, Islam et al. (2008) showed that chronic adenylyl cyclase (AC) and cAMP signaling increase the release of vesicle-bound tumor necrosis factor receptor 1 (TNFR1), measured during long-term culture. Using our CD63-pHluorin reporter, we observed that these agents also stimulate MVB–PM fusion (Fig. S3 d). GPCRs are attractive drug targets that signal via cAMP, IP3, and DAG, activating G proteins that in turn activate AC/PLC (Simon et al., 1991). Histamine is a ligand for GPCRs expressed in tumors (Falus et al., 2010) and a stimulant of HeLa cells (Smit et al., 1992). We stimulated single HeLa cells with histamine via a glass capillary positioned right above the cover slide at the border of the imaging window, a setup known as a superfusion system (Fig. 4 a). Superfusion of 100 µM histamine immediately increased the rate of MVB–PM fusion events, whereas this fusion burst was not observed with KCl nor caffeine-induced release of calcium from internal stores (Fig. 4, b and c). Despite the diversity of basal fusion activity between cells, we recorded consistent induction after histamine stimulation (Fig. 4 d). Thus, histamine-induced GPCR signaling appears to be coupled to inducible MVB–PM fusion.

Because many GPCRs use a Ca^2+^ secondary messenger system (Dickenson and Hill, 1994), we monitored the MVB–PM fusion rate upon histamine stimulation while simultaneously measuring intracellular calcium levels. Unlike ultrafast calcium-induced synaptic vesicle release in neurons (Südhof, 2013), we observed a delay of ∼10 s between calcium entry and increased MVB–PM fusion activity (Fig. 4 e). In fact, addition of the fast Ca^2+^ buffer BAPTA-AM (20 µM), an intracellular calcium chelator, or the slow buffer EGTA-AM (200 µM), an extracellular calcium chelator, did not significantly reduce histamine-induced MVB–PM fusion in HeLa cells (Fig. 4, f and g). Thus, histamine-induced MVB–PM fusion in HeLa cells does not seem to require an immediate Ca^2+^ influx nor release from cytosolic storage compartments, unlike secretory lysosomes (Rodríguez et al., 1997). To extend our findings to nontransformed cells, we stimulated CD63-pHluorin HUVEC cells with the same protocol of 100 µM histamine for 1 min. Although the basal fusion rates are lower than in HeLa cells, we observed a significant increase in fusion activity in single cells after stimulation with histamine (Fig. 4 h), suggesting that MVB–PM fusion can be modulated by external cues in both primary and transformed cells.

### SNAP23 is a GPCR downstream effector regulating constitutive exosome secretion

**Figure 5.**
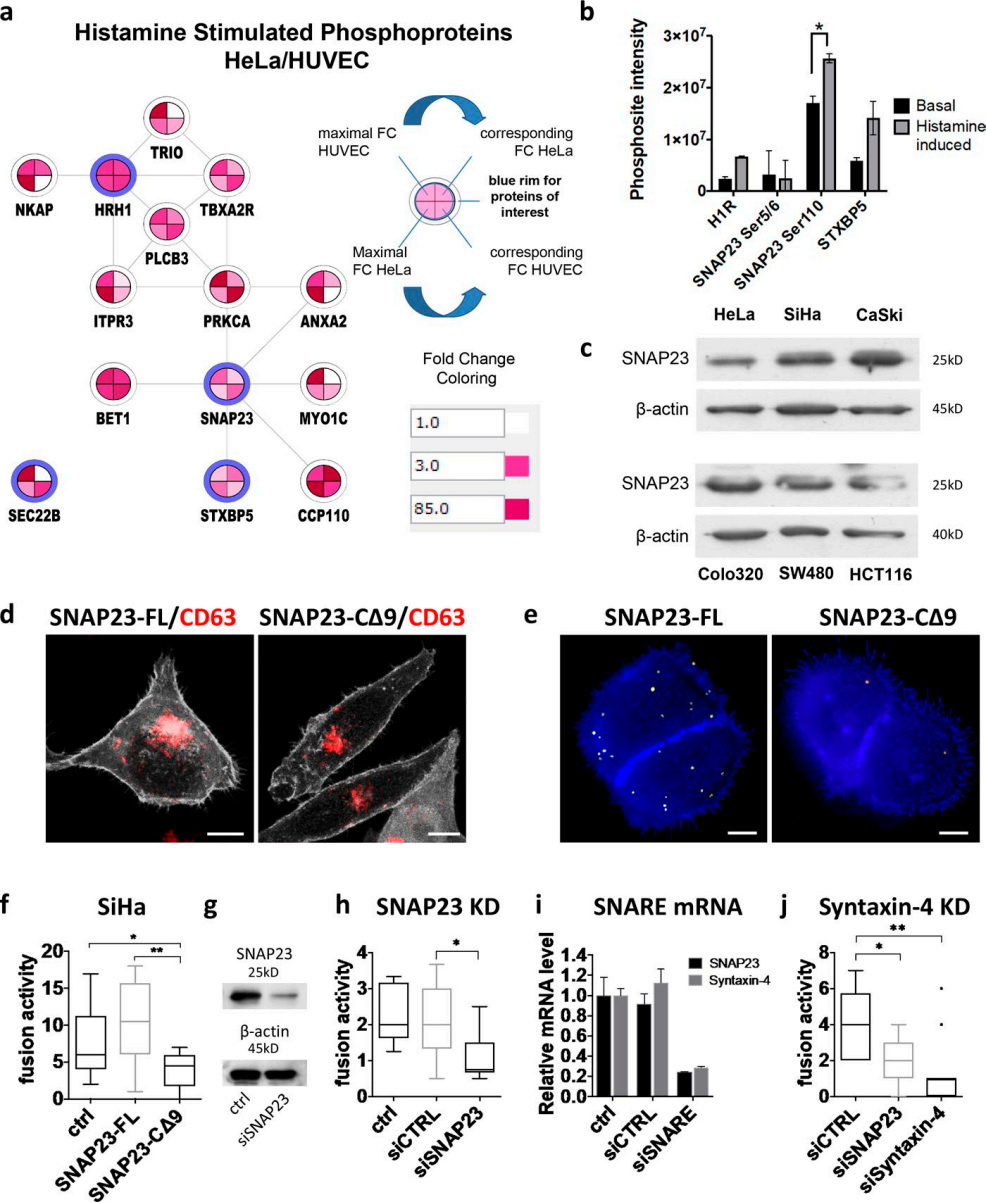
**The GPCR downstream effector SNAP23 regulates MVB–PM fusion. (a)** Network of proteins of interest together with direct interactors with altered phosphorylation levels upon histamine (100 µM) stimulation as identified by phosphoproteomics in HeLa and HUVEC cells. Proteins of interest are depicted with a blue rim. FC, fold change. **(b)** Graph showing the signal intensity values of phosphorylated peptides from proteins of interest before and after stimulation with 100 µM histamine. Data represent means ± SD of two technical replicates per condition. *, P < 0.05 (P = 0.048) using Student’s two-tailed two-sample *t* test. **(c)** Western blotting analysis on SNAP23 protein expression in six different cell lines. **(d)** Confocal analysis of FL (GFP-SNAP23-FL) and truncated (GFP-SNAP23-CΔ9) GFP-SNAP23 (in gray)–transfected SiHa cells labeled for CD63 (red). **(e)** Total projection of fusion events in CD63-pHluorin SiHa cells cotransfected with SNAP23-FL or SNAP23-CΔ9 over 3 min. Pseudocolored as in Fig. 1 j. Bars, 10 µm. **(f)** Quantification of fusion events in CD63-pHluorin SiHa cells cotransfected with SNAP23-FL or SNAP23-CΔ9. *n* ≥ 10 cells per condition. **(g)** Confirmation of SNAP23 knockdown (KD) at the protein level in HeLa cells. **(h)** Effect of SNAP23 knockdown on MVB–PM fusion in HeLa cells. *n* ≥ 17 cells per condition. **(i)** Confirmation of SNAP23 and syntaxin-4 knockdown in HeLa cells at the mRNA level. Data represent means ± SD. **(j)** Effect of the knockdown of SNAP23 or syntaxin-4 on the fusion activity of HeLa cells. *n* ≥ 11 cells per condition. ctrl, nontransfected; siCTRL, control siRNA. *, P < 0.05; **, P < 0.01 using Student’s two-tailed two-sample *t* test. Whiskers in the box plots (f, h, and j) represent 1.5 times the interquartile distance or the highest or lowest point, whichever is shorter.

To search for target proteins that could link histamine stimulation to the imminent induction of MVB fusion with the PM, we performed phosphoproteomics on HeLa and primary HUVECs after 1 min of 100 µM histamine stimulation (Table S3). Subsequent computational analysis identified an activated protein network that connects the H1HR to SNAP23, protein kinase C α (PKCα), and additional direct and indirect interactors (Figs. 5 a and S4 a). Gene Ontology (GO) terms for this extended network included “plasma membrane,” “cytoplasmic membrane–bounded vesicle,” “vesicle fusion,” and “vesicle-mediated transport,” thus compatible with exosome physiology (Fig. S4 b). Consistent with this, histamine stimulation increased phosphorylation of the membrane fusion proteins STXBP5 and SNAP23 (Fig. 5 b and Table S1).

Among the potential downstream effectors of histamine stimulation, SNAP23 as a member of the SNARE protein family involved in membrane fusion processes was of prime interest. We found that the exocytic t-SNARE SNAP23 was expressed in six different cell lines (Fig. 5 c) and enriched in exosomes as determined with quantitative proteomics (Table S2; Baglio et al., 2016). For functional assays, we used a C-terminal truncated form of SNAP23 (CΔ9) that competes with the endogenous SNAP23 and forms nonfunctional complexes with SNARE partners (Kawanishi et al., 2000). Confocal analysis indicated that exogenous WT SNAP23 and mutant SNAP23-CΔ9 both localize to the PM (Fig. 5 d; Kean et al., 2009). When these constructs were coexpressed with CD63-pHluorin, SNAP23-CΔ9–expressing cells had reduced MVB–PM fusion activity compared with cells expressing exogenous WT SNAP23 (–full-length [FL]) or nontransfected cells (Fig. 5, e and f). Importantly, the decrease in MVB–PM fusion activity caused by SNAP23-CΔ9 expression corresponded with a significant decrease in secreted exosomes as determined by Western blotting for CD63 and CD81 (Fig. S4 c).

To show direct involvement of SNAP23 protein in MVB–PM fusion, we generated SNAP23-depleted cells using siRNA (Fig. 5 g) and expressed CD63-pHluorin in these cells. Live imaging revealed that depletion of SNAP23 reduced the MVB–PM fusion rate (two- to threefold) compared with control siRNAs (Fig. 5 h). Moreover, depletion of syntaxin-4, a functional partner of SNAP23 in exocytosis (Kawanishi et al., 2000), lead to comparable inhibition of MVB–PM fusion activity (Fig. 5, i and j). These studies suggest that the SNAP23–syntaxin-4 SNARE complex regulates MVB–PM fusion and TSPAN-enriched exosome release from HeLa cells.

### Histamine-induced MVB–PM fusion is triggered by phosphorylation of SNAP23

**Figure 6.**
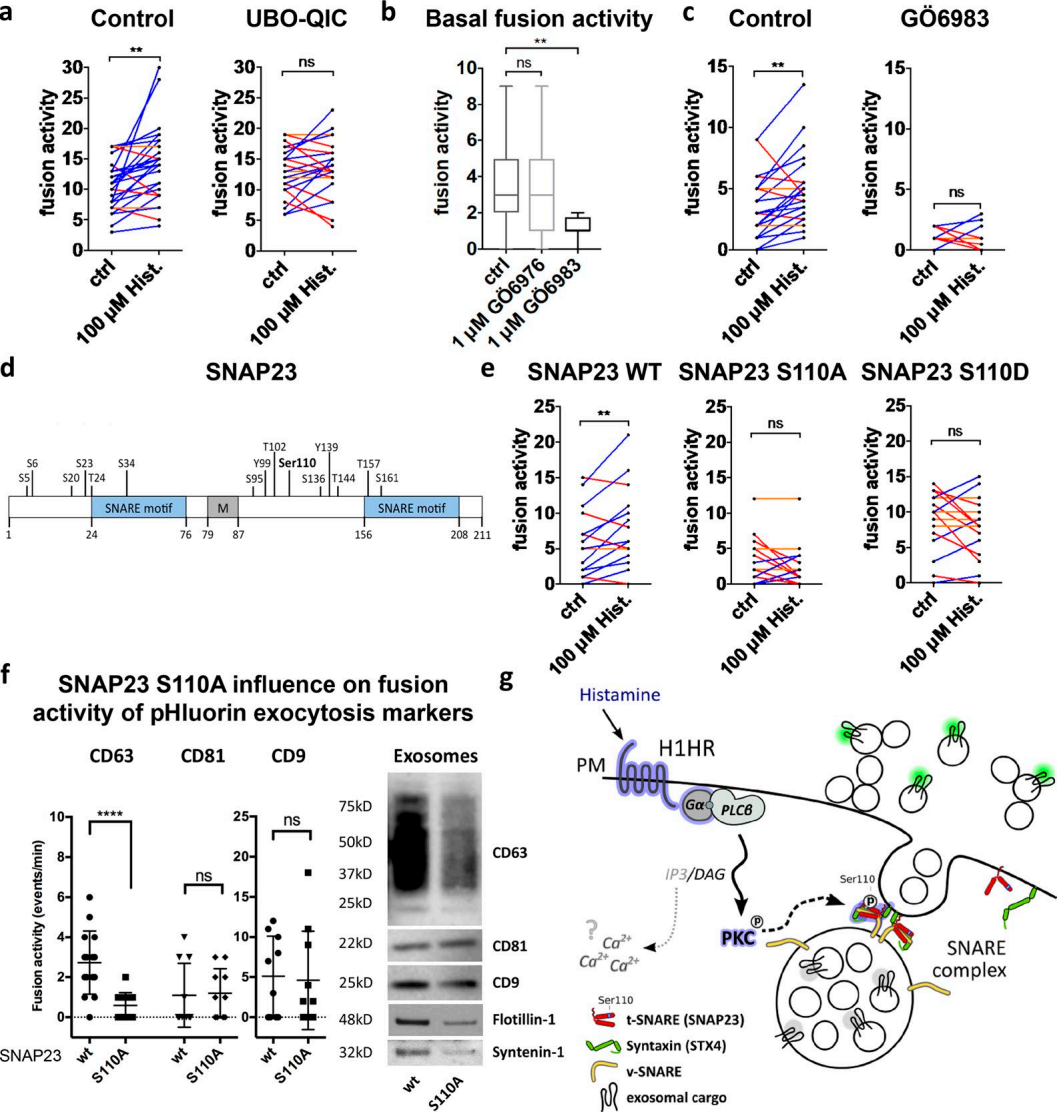
**GPCR activation triggers MVB–PM fusion in HeLa cells via SNAP23-Ser110 phosphorylation. (a)** Fusion activity of histamine-stimulated cells nontreated or treated with the Gα_q_ inhibitor UBO-QIC (1 µM). *n* ≥ 24 cells per condition. **(b)** Basal fusion activity in cells treated with PKC inhibitors GÖ6976 (1 µM) or GÖ6983 (1 µM). *n* ≥ 11 cells per condition. **(c)** Fusion activity of histamine-stimulated cells nontreated or preincubated with GÖ6983 (1 µM). *n* ≥ 11 cells per condition. **(d)** Schematic representation of SNAP23 with SNARE motifs, a membrane-anchoring domain (M), and all phosphosites with the posphosite targeted by histamine stimulation (Ser110) in bold. **(e)** Fusion activity of histamine-stimulated cells transfected with WT SNAP23, phosphomutant SNAP23-S110A, or phosphomimic SNAP23-S110D. *n* ≥ 16 cells per condition. **(f)** Left: fusion activity of CD63-, CD81-, and CD9-pHluorin HeLa cells cotransfected with SNAP23 WT or SNAP23-S110A. *n* ≥ 16 cells per condition. Western blot on exosomes isolated from SNAP23-WT and SNAP23-S110A HeLa cells labeled for CD63, CD9, CD81, flotillin-1, and syntenin-1. **(g)** Schematic representation of the histamine-stimulated pathway leading to exosome release as identified by phosphoproteomics and specific inhibitors. Blue-rimmed proteins represent the putative pathway implicated by both experiments. The IP3–Ca^2+^ pathway is represented in gray as a direct link with MVB–PM fusion is missing. **, P < 0.01; ****, P < 0.0001 using Student’s two-tailed two-sample *t* test. All *t* tests were paired except for b and f. Whiskers in the box plots in b and f represent 1.5 times the interquartile distance or the highest or lowest point, whichever is shorter.

To identify the mechanism for regulated exosome secretion, we postulated that histamine signaling induces MVB–PM fusion through phosphorylation of SNAP23 complex members (Fig. 5 a). The histamine receptor (H1HR) couples Gα_q_ subunits to phospholipase C, resulting in IP3 and DAG production and activation of PKC. Fittingly, pretreatment of HeLa cells with the specific Gα_q_ inhibitor UBO-QIC significantly reduced the stimulatory effect of histamine on MVB–PM fusion rate (Fig. 6 a), consistent with Gα_q_ and the H1HR pathway causing activation of SNAP23 by phosphorylation. To verify the possible involvement of PKC upstream of SNAP23 phosphorylation in histamine-induced MVB–PM fusion, we blocked its kinase activity with PKC inhibitors GÖ6983 (1 µM) and GÖ6976 (1 µM). Incubation with the pan-PKC inhibitor GÖ6983, but not the PKCα/β-selective GÖ6976, significantly reduced basal MVB–PM fusion activity (Fig. 6 b). Moreover, GÖ6983 treatment abrogated the stimulatory effect of histamine on MVB–PM fusion, suggesting a key role for nonconventional calcium-independent PKC in histamine-induced MVB–PM fusion (Fig. 6 c).

SNAP23 has a role in diverse membrane fusion processes, and phosphorylation of defined serine residues has been implicated in distinct exocytic processes (Fig. 6 d; Polgár et al., 2003; Karim et al., 2013) including recently constitutive exosome release (Wei et al., 2017). Our phosphoproteomic analysis on HeLa cells revealed phosphorylation of SNAP23 at Ser5/6 and Ser110, whereas Ser95 was not detected in our screen (Fig. 5 b). Importantly, histamine-induced SNAP23 phosphorylation occurred specifically at serine residue 110, a phosphorylation site of SNAP23 with a poorly understood function. To investigate whether Ser110 has a role in MVB–PM fusion, we modified serine 110 of SNAP23 into an alanine (S110A) to create a phosphomutant and into an aspartic acid residue (S110D) to create a phosphomimetic version. Whereas expression of the phosphomimic SNAP23-S110D displayed a similar CD63-pHluorin fusion activity as WT SNAP23, expression of SNAP23-S110A significantly decreased the basal fusion activity of CD63-pHluorin without altering the localization of SNAP23 (Figs. 6 e and S4 d). Moreover, both SNAP23-S110A and -S110D abrogated histamine-induced MVB–PM fusion (Fig. 6 e). In contrast with CD63-pHluorin, CD81-pHluorin and CD9-pHluorin fusion events were not affected by SNAP23-S110A expression. Strikingly, we found that SNAP23-S110A expression reduced the amount of CD63 exosomes as confirmed with flotillin-1 and syntenin-1 by Western blotting, whereas CD81 and CD9 appear less reduced (Fig. 6 f). Collectively, our results are consistent with a model in which histamine-regulated MVB fusion with the PM requires phosphorylation of SNAP23 at serine 110 that drives the externalization of CD63-enriched exosomes (Fig. 6 g).

## Discussion

Apart from soluble factors, most, if not all cell types, release EVs including MVB-derived exosomes with physiological functions in development, immune responses, and pathology (Peinado et al., 2012). Much insight originated from analyzing the biochemical composition of total EVs and subtypes isolated from cell culture supernatants and biofluids using ultracentrifugation and sucrose density gradients. Although the dynamics of exosome biogenesis and release have remained elusive, these aspects may be equally important for their physiological role in vivo. In this study, we designed a live single-cell imaging approach to monitor MVB–PM fusion and exosome release from living mammalian cells under variable conditions. We found that activation of a GPCR signaling pathway leads to rapid phosphorylation of the t-SNARE SNAP23 at serine 110 (Ser110), activating the MVB–PM fusion machinery required for the release of CD63-enriched EVs.

To study the kinetics of exosome release, we designed and validated CD63-pHluorin as an optical reporter to monitor MVB–PM fusion events over time. Our approach allowed for quantification of defined subpopulations of exosome release from single cells and enabled us to study the effects of rapid and temporary changes in the local environment, which is a major advance over standard biochemical methods. Despite these advantages, our imaging approach ignores generation of MVs or exosome-like vesicles that bud from the PM, which could be viewed as a limitation (Colombo et al., 2014). Also, EV subpopulations devoid of the chosen reporter will not be visible. Vesicle release may also be overestimated in that our approach cannot distinguish MVB–PM fusion from fusion of acidic MVBs with neutral vesicles that may be adjacent to the PM and/or because MVB–PM fusion may not lead to actual vesicle release into the extracellular space as they remain stuck at the PM (Fig. 1 d; Edgar et al., 2016). Finally, because a proportion of late endo/lysosomal CD63 will traffic to the cell surface (Latysheva et al., 2006) or remain at the limiting membrane of MVBs as showed by our EM data and that of others (Escola et al., 1998; Verweij et al., 2011), part of the fluorescent signals will be derived from CD63-pHluorin present on these locations.

Despite these considerations, our imaging results with the C-terminal CD63-pHluorin reporter demonstrate that the majority of CD63-pHluorin fusion events signify externalization of ILVs as exosomes (Fig. S2, a and b). This conclusion is further supported by our dynamic CLEM results, which unequivocally demonstrate that CD63-pHluorin–associated bursts mark true MVB–PM fusion spots where ILVs are externalized as exosomes, as originally proposed by Harding et al. (1983). In addition, we could map exosome secretion at a subcellular scale. The use of poorly migrating/invading cell types in this study may explain why we could not observe fusion events at sites of adhesion assembly as others have already reported (Sung et al., 2015). Finally, we could show that a reduction in MVB–PM fusion activity as determined by live imaging leads to lower concentrations of TSPAN-enriched EVs in the supernatant as determined by Western blotting after differential ultracentrifugation (Fig. 1, k and l; Fig. 6 f; and Fig. S4 c).

Apart from the frequency of fusions, we found that the nature of the fusion events provides qualitative information. When we compared the signal duration of CD63-pHluorin fusion events with VAMP2/NPY reporters, we observed that CD63-pHluorin vesicles are distinct from general transport vesicles fusing with the PM (Fig. 3, d and g). The prolonged burst of fluorescence may be explained by reduced dispersion of externalized fluorescent exosomes in the medium when compared with soluble cargo release (NPY-pHluorin) and/or lateral membrane diffusion combined with endocytosis of PM-deposited VAMP2-pHluorin. Although CD63/CD81 fusion events are similar in duration, CD9 was significantly shorter and more similar to VAMP2, suggesting membrane deposition rather than exocytosis. In fact, a CD9 subpopulation of small EVs has been identified that lacks CD63 and CD81 and may represent EV budding from the PM (Kowal et al., 2016). The divergence in fusion duration could thus reflect a difference between endosome–PM fusion for CD63/CD81-pHluorin and fusion of Golgi-derived CD9/VAMP2/NPY-pHluorin–positive transport vesicles with the PM. Applying the CLEM technique (as in Fig. 2) with the different pHluorin-reporters could yield more insights in future studies.

Despite the decidedly different methods, we were able to correlate live imaging quantitation of MVB–PM fusions to standard biochemical procedures for quantifying EV release. We found that reduced nSMase-2 activity or dysfunctional SNAP23 complexes diminish basal MVB–PM fusion activity, corresponding with a reduced amount of CD63-enriched EVs (Fig. 1, k and l; Fig. 6 f; and Fig. S4 c). Interestingly, although GW4869 treatment and SNAP23-CΔ9 led to a general reduction of EVs for CD63 and CD81, expression of SNAP23-S110A seemed to have its greatest effect on CD63-enriched EV release. It is tempting to speculate that defined phosphorylation sites on the exocytic t-SNARE SNAP23 can mediate the release of exosome subtypes. However, these results need to be interpreted with caution as we cannot formally rule out effects of SNAP23-S110A on TSPAN expression or sorting, even though the broader-acting SNAP23-CΔ9 mutant does not affect CD63 localization (Fig. 5 d).

A requirement of different fusion mechanisms at target membranes for distinct subtypes of MVBs was originally proposed by Simons and Raposo (2009). Our results seem to indicate a specificity for SNAP23-Ser110 on the MVB subtype level, whereas Wei et al. (2017) implicated Ser95 in constitutive release of the total pool of EVs, mediated by PKM2 signaling. Future studies could benefit from the various TSPAN reporters used in this study to clarify whether differentiation based on phosphorylation of distinct SNAP23-serine residues is a general mechanism or whether more regulatory mechanisms exist, e.g., on other SNARE complex partners such as Munc18-1 (Toonen and Verhage, 2007).

The phosphoproteomic screen indicated phosphorylation of H1R1 that mediates histamine signaling in HeLa cells (Smit et al., 1992). We therefore suggest that the H1HR–Gα_q_–PKC pathway rather than the AC–cAMP–PKA pathway stimulates MVB–PM fusion in HeLa cells (Fig. 6 g). Indeed, inhibitors of Gα_q_ and PKC attenuated histamine-induced MVB–PM fusion in these cells (Fig. 6, a–c). Although cells in culture have no cell polarity and seem to have a constitutive and overactive MVB–PM fusion profile, regulated and localized release of exosomes in vivo may have important biological consequences. In fact, the immunological synapse (IS) between B and T cells is one of the few examples where the physiological context for regulated exosome secretion was investigated (Mittelbrunn et al., 2011). A functional IS requires the recruitment of the GPCRs CCR5 and CXCR4 for T cell activation (Contento et al., 2008) that coincides with localization of PKCα in the IS (Gharbi et al., 2013), suggesting that polarized secretion of exosomes can be triggered by similar pathways. But how could such events be controlled mechanistically?

We demonstrated with a phosphomutant and phosphomimetic of SNAP23 (S110A/S110D) that histamine-stimulated and constitutive MVB–PM fusion requires functional SNAP23 complexes (Fig. 6 e). Presumably, basal levels of phosphorylated Ser110 of endogenous SNAP23 are present before the histamine stimulus, which are competed away by SNAP23-S110A (Fig. 5 b). Our observations do not, however, exclude the involvement of other phosphorylation sites of SNAP23 in MVB–PM fusion such as Ser95 (Wei et al., 2017). However, in our screen with HeLa and HUVEC cells, Ser110 was the only phosphorylation site of SNAP23 that was increased by histamine stimulation and of which the kinetics matched the increase in MVB–PM fusion (Fig. 5 b). It would be of interest for future studies to determine whether physiological differences exist between constitutively released exosomes and those that are released upon GPCR stimulation.

In this study, we characterized different TSPAN-pHluorin reporters and explored exogenous triggers, intracellular signaling pathways, and a t-SNARE directly involved in the release of exosomes. The advance of this live-imaging approach is that effects of interfering agents can be visualized in real-time, minimizing the risk of measuring secondary effects, and could constitute an interesting potential for small targeted screens. An additional strength of this tool is that it also allows to study the physiology of exosomes in more detail on a single-cell basis, e.g., by studying exosome release at invadopodia (Hoshino et al., 2013). In summary, with live and correlative imaging, we identified previously unknown modulators of MVB–PM fusion and exosome release in living single cells. Our approach offers new avenues for understanding the physiological function of exosome secretion in vitro and in vivo and to translate this knowledge into potential targets for modulating this process.

## Materials and methods

### Cell lines

HeLa, SiHa, HCT116, and SW480 cells were cultured in DMEM supplemented with 10% FBS (Perbio Sciences; HyClone), 100 U/ml penicillin G, 100 mg/ml streptomycin sulfate, and 2 mM glutamine. Tetherin knockout cells were a gift from J. Edgar (University of Cambridge, Cambridge, England, UK). HUVEC cells were cultured in EGM-2 medium (Lonza) supplemented with 2% FBS. Adipose-derived human MSCs were cultured in α-MEM supplemented with 10% FBS, 100 U/ml penicillin G, 100 mg/ml streptomycin sulfate, and 2 mM glutamine.

### Plasmids

The pCMV-CD63-pHluorin plasmid was generated by modifying the pCMV-CD63 plasmid, a gift from K. Sato (Kyoto University, Kyoto, Japan). We first amplified the superecliptic pHluorin sequence out of a synapto-pHluorin plasmid (Miesenböck et al., 1998) using the primer pair 5′-TAGCTAGATCTATGGGAAGTAAAGGAG-3′ and 5′-TCGCTAGATCTTTTGTATAGTTCATCCAT-3′ to add BglII restriction sites at both ends of the pHluorin sequence. Next, we generated pCMV-CD63×ECI by inserting a BglII restriction site in the ECL1 of CD63 between amino acids Gln36 and Leu37 by targeted mutagenesis of pCMV-CD63 using QuikChange Lightning Site-Directed Mutagenesis kit protocol (Agilent Technologies) and 5′-GTGTCGGGGCACAGAGATCTCTTGTCCTGAGTCA-3′ as a primer. The final pCMV-CD63-pHluorin was obtained by ligating the pHluorin insert with the mutated pCMV-CD63×ECI vector after digestion with BglII, generating pCMV-CD63-pHluorin. The pCMV-CD81-pHluorin and pCMV-CD9-pHluorin were constructed following the same strategy. The original CD81- and CD9-encoding constructs that served as templates were gifts from A.B. van Spriel (Radboud Institute for Molecular Life Sciences, Nijmegen, Netherlands). For CD63–C-term–pHluorin, the BglII site was inserted before the stop codon of CD63 by PCR using the primers 5′-ATATCTAGACTTAAGATCTCATCACCTCGTAGCCACTTCTG-3′ and 5′-ATAGAATTCCATGGCGGTGGAAGGAG-3′. The VAMP2-pHluorin and NPY-pHluorin were gifts from J. Rothman (Yale University, New Haven, CT) and R. Holz (University of Michigan, Ann Arbor, MI). pDisplay-pHuji was a gift from R. Campbell (University of Alberta, Edmonton, Canada; 61556; Addgene). pHuji was amplified from pDisplay-pHuji by PCR with 5′-TAAGAAGATCTATGGTGAGCAAGGGCGAGGAGAATAAC-3′ and 5′-TAAGAAGATCTCTTGTACAGCTCGTCCATGCCGC-3′ to add BglII restriction sites at both ends. The GFP-SNAP23 FL and C-terminally truncated SNAP23 (GFP)SNAP23-CΔ9 plasmids have been described previously (Kean et al., 2009). SNAP23-S110A was constructed by Gibson assembly using primer pairs 5′-ACAACATGGGGAGATGGTGGAGAAAACGCTCCTTGC-3′ and 5′-TTTAGATACTACATTGCAAGGAGCGTTTTCTCCACC-3′, and SNAP23-S110D using primer pairs 5′-ACAACATGGGGAGATGGTGGAGAAAACGATCCTTGC-3′ and 5′-TTTAGATACTACATTGCAAGGATCGTTTTCTCCACC-3′.

### Verification of CD63-pHluorin trafficking

We positioned a superecliptic pHluorin moiety into the first external loop of CD63, an integral membrane protein enriched in ILVs of MVBs in many cell types (Escola et al., 1998; Verweij et al., 2011). Confocal imaging of fixed HeLa cells expressing CD63-pHluorin showed a near-complete overlap with endogenous CD63 (Pearson coefficient, 0.96). Although our antibody most likely recognized both endogenous CD63 and CD63-pHluorin, single-red vesicles, indicative of a separation between the exogenous CD63-pHluorin and endogenous CD63 pools, were not detected, suggesting that CD63-pHluorin is not subject to trafficking defects (Fig. 1 b). As the antibody used was likely conformational, we expected that the conformation of ECL2 was largely preserved. Next, we neutralized acidic compartments in living CD63-pHluorin–expressing HeLa cells with NH_4_Cl and found that a significant proportion (70–90%) of CD63-pHluorin resided in acidic vesicles, turning fluorescent upon NH_4_Cl exposure (Fig. 1 c). Finally, observation by EM of immunogold-labeled ultrathin cryosections of CD63-pHluorin–expressing cells (immuno-EM) confirmed the efficient sorting of CD63-pHluorin into ILVs of MVBs (Figs. 1 d and S1, a and b).

### Transfections

Plasmid transfections were usually performed using Lipofectamine 2000 reagent (Invitrogen) or JetPRIME (Polyplus) typically on a 12-well scale with 500 ng plasmid and cells at 50–70% confluency. Cells were examined after 24-h transfection. Adipose-derived human MSCs were microporated using the Neon transfection system (Invitrogen) with 0.5 µg of a plasmid according to the manufacturer’s protocol using the following settings: three pulses at 1,400 V with 10-ms pulse width. HUVEC cells were transfected with 1 µg DNA per well (12-well plates) using 3 µl jetPEI-HUVEC (Polyplus).

### RNA interference

HeLa cells were seeded on poly-l-lysine–coated coverslips (18 mm; Thermo Fisher Scientific) at 20,000 cells per well in 1 ml of complete growth medium 24 h before siRNA transfection. Cells were transfected with 5 µl of 5 µM ON-TARGETplus SMARTpool or 7.5 µl of 100 µM Accell SMARTpool siRNA (GE Healthcare) according to the manufacturer’s protocol. For MVB fusion imaging purposes, cells were transfected with the CD63-pHluorin plasmid 48 h after siRNA transfection and assessed the next day.

#### siRNA sequences

SNAP23 ON-TARGETplus SMARTpool (L-016256-00-0005): 5′-CAUUUGUUGAGUUCUGUUA-3′, 5′-CUUCUAUGCGGUUUAGUUG-3′, 5′-AUAUCAAUACGAUCUCUGU-3′, and 5′-UUAGCUGUCAAUGAGUUUC-3′; SNAP23 Accell SMARTpool (E-017545-00-0005): 5′- AGAUUUCCCAGGAUACUGC-3′, 5′-GAGACUCAAUGGCUAAACC-3′, 5′-UCCUAACUAAACAUAAUUC-3′, and 5′-ACAUAGUUCAAUACACAAG-3′; syntaxin-4 ON-TARGETplus SMARTpool (L-016256-00-0005): 5′-AUAGUCUGCCGAAUUGUCC-3′, 5′-UAUCCAACCACUGUGACGC-3′, 5′-UUGGACACAAACACCUCGC-3′, and 5′-UCUCAUUCUUGUGUUGACG-3′; and nSMase2 Accell SMARTpool (E-006678-00-0005): 5′-UAGCAUGGAAAGAUAAGGG-3′, 5′-UCAAUUUGGUGGCUGCUCG-3′, 5′-AAAGAACCCUGGACGAAGC-3′, and 5′-AACCUUCGCUGCAUGACAG-3′.

### Antibodies and reagents

Monoclonal antibodies against CD63 (mouse; H5C6; 556019; BD), CD81 (mouse; JS-81; 551112; BD), CD9 (mouse; a gift from E. Rubinstein, Institut National de la Santé et de la Recherché Médicale, Villejuif, France), and Alix (3A9; 2171; Cell Signaling Technology) were used at 1:200. Syntenin-1 (rabbit; 610821; BD) and flotillin-1 (rabbit; ab19903; Abcam) mouse antibodies were used at 1:250. Antibodies against SNAP23 (rabbit; 111-202; Synaptic Systems), GFP (rabbit; A01388; Genscript), and β-actin (mouse; sc-47778; Santa Cruz Biotechnology, Inc.) were used at 1:1,000. Secondary anti–mouse Alexa Fluor 594 antibody (goat; A-11032; Invitrogen) was used at 1:500. For Western blotting, cells or exosomes were lysed in a 1% SDS buffer, and equal amounts of protein were loaded onto an SDS-PAGE gel. Only gels for CD63, CD81, and CD9 detection were run under nonreducing conditions.

EGTA-AM (Sigma-Aldrich) was applied at 200 µM for 15 min at 37°C, BAPTA-AM (Sigma-Aldrich) was applied at 20 µM for 15 min at 37°C, and GÖ6983 (Axon Medchem) and GÖ6976 (EMD Millipore) were applied at 1 µM for 2 h at 37°C. GW4869 (Sigma-Aldrich) was applied at 5 µM for 4 h. Histamine (Sigma-Aldrich) was used at 100 µM, and caffeine (Sigma-Aldrich) was used at 20 mM. UBO-QIC (a gift from E. Kostenis, University of Bonn, Bonn, Germany) was used at 1 µM for at least 30 min (preincubation).

### Image acquisition and data analysis

#### Dual-TIRF microscopy

Coverslips were placed in an imaging chamber and perfused with Tyrode’s solution (2 mM CaCl_2_, 2.5 mM KCl, 119 mM NaCl, 2 mM MgCl_2_, 20 mM glucose, and 25 mM Hepes, pH 7.4). All videos except for those used for Fig. 6 (e and f) and Fig. S2 b were imaged on a microscope (Axiovert 200M; ZEISS) equipped with an electron-multiplying charge-coupled device camera (CASCADE; Roper Scientific) and an illumination unit (Polychrome IV; TILL Photonics) for widefield imaging using a 40× 1.3 NA widefield objective. For TIRF imaging, a laser beam from an air-cooled argon ion laser was coupled into a 100× 1.45 NA TIRF objective via a TIRF condenser (TILL Photonics). Images were acquired at 2 Hz unless noted otherwise and were acquired with MetaMorph 6.2 software (Universal Imaging; Molecular Devices). The histamine-stimulation protocol consisted of 3 min imaging in total, including 1 min superfusion with 100 µM histamine in Tyrode’s solution. Intracellular pH was neutralized with normal Tyrode’s solution containing 50 mM NH_4_Cl instead of NaCl. A barrel pipette (ALA-VM4) was used to apply NH_4_^+^ solution to the cells. All imaging experiments were performed at RT (21–24°C).

For Fig. 6 (e and f) and Fig. S2 b (dual-TIRF), a T*i*-E inverted microscope setup was used with a 100× CFI Apochromat TIRF, oil, 1.49/0.12 mm, a/0.17 differential interference contrast objective (Nikon) and a laser bench (Roper Technologies). MetaMorph software was used for all acquisitions.

#### Dynamic CLEM and electron tomography

HeLa cells were seeded on gridded coverslips (MatTek) and transfected with CD63-pHluorin–encoding plasmids 24 h later. Live-cell imaging experiments were performed 24 h after transfection at an inverted spinning-disk microscope (T*i*-E infrared microscope [Nikon] equipped with a CSU-X1 lens [Yokogawa Electric Corporation]) at RT using an oil-immersion Plan Apochromat 100× 1.40 NA objective lens and an electron-multiplying charge-coupled device 512 × 512 Evolve camera (Photometrics). Under the microscope, a mixture of 2.5% PFA + 0.125% glutaraldehyde in 1 mM phosphate buffer, pH 7.4, was added on cells at the appearance of burst of fluorescence. Fixative buffer was exchanged for 2.5% glutaraldehyde in 0.1 M cacodylate buffer for 24 h and post-fixed with 1% osmium tetroxide, dehydrated in ethanol, and embedded in Epon (Raposo et al., 2001). The first sections of cell monolayers of 250-nm thickness and corresponding with the interface between the cell and the coverslip were prepared with a Reichert UltracutS ultramicrotome (Leica Microsystems) and were randomly labeled on both sides with 15 nm Protein A Gold and then post-stained with uranyl acetate lead citrate. All samples were examined with a Tecnai Spirit electron microscope (FEI Company), and digital acquisitions were made with a numeric camera (Quemesa; Soft Imaging System). Identification of the region of interest and estimation of the correlation error range were performed using eC-CLEM (Paul-Gilloteaux et al., 2017).

Tomographic acquisitions of the regions of interest were made on 250-nm-thick sections. Tilt series (angular range from −60° to +60° with 1° increments) were recorded by using Xplore3D (FEI) on 200-kV transmission electron microscopes (Tecnai 20 LaB6; FEI). Images (1,024 Å ∼ 1,024 pixels) were recorded using an F416 CMOS 4,000 × 4,000 pixel camera (TVIPS). Tilt series alignments and weighted back-projection reconstructions were performed using eTomo (IMOD) software. 15 nm Protein A Gold at the surface of the sections was used as fiducial marker. Manual contouring of the tomograms was done using IMOD program.

#### Imaging flow cytometry

Cells were acquired on an ImageStreamX imaging flow cytometer (Amnis) using a 488-nm laser set at 80 mW. A minimum of 2,000 cells was acquired per sample at 60× magnification at a flow rate ranging between 25 and 50 cells per second. At this magnification, lateral resolution was ∼250 nm, whereas the depth of the optical slice was ∼4 mm (Bolte and Cordelières, 2006). Analysis was performed using the IDEAS software (6.0; Amnis). A compensation table was generated using the compensation macro built in the software and applied to the single staining controls. Proper compensation was then verified by visualizing samples in bivariate fluorescence intensity plots. A template analysis file to gate for single optimally focused cells was prepared and applied to the experimental samples in order to export this population to a new compensated image file to allow merging all experimental samples within a single file for direct sample analysis. The peak mask was used to detect individual endosomes by setting a signal/noise ratio of 8. Then, the number of endosomes was calculated using the feature spot count on the previously computed endosomal (peak) mask.

#### Confocal microscopy

For confocal laser scanning microscopy (CLSM) analysis, cells were seeded on poly-l-lysine–coated ø 18-mm coverslips, fixed with 4% PFA (20 min), permeabilized with 0.1% Triton X-100 (10 min), and blocked with PBS containing 10% FCS (1 h). The slides were incubated with CD63 primary antibody for 30 min at RT followed by incubation with anti–mouse Alexa Fluor 594 secondary antibody for 30 min at RT, mounted with the Vectashield reagent (Vector Laboratories), and sealed with nail polish. CLSM was performed at RT on a TCS SP8 X microscope (Leica Microsystems). Samples were irradiated with a pulsed white light laser at 488 or 594 nm. The signals were detected using a gated Hybrid Detector (HyD; Leica Microsystems). CLSM images were acquired using an oil objective with 63× magnification and 1.4 NA. Application Suite software (Leica Microsystems) was used for image acquisition. ImageJ (National Institutes of Health) software was used for statistical analysis, including calculation of Pearson’s correlation coefficients.

#### Immunoelectron microscopy

HeLa cells were fixed for 2 h in a mixture of 2% PFA + 0.2% glutaraldehyde in 60 mM Pipes, 25 mM Hepes, 2 mM MgCl_2_, and 10 mM EGTA, pH 6.9, and then processed for ultrathin cryosectioning. The isolated exosomes were spotted on a glow-discharged formvar-coated grid and fixed with 4% PFA to perform immunoelectron microscopy. For immunolabeling, the sections/exosomes were incubated for 10 min with 0.15 M glycine in PBS and for 10 min with 1% BSA in PBS to block free aldehyde groups and prevent aspecific antibody binding, respectively. Sections were incubated with anti-GFP antibody followed by 10 nm protein A–conjugated colloidal gold (EMlab; University of Utrecht), all in 1% BSA in PBS. Next, the cryosections/exosomes were embedded in uranylacetate and methylcellulose and examined with a CM 10 electron microscope (Philips Healthcare) equipped with a flat film camera (FEI).

#### Imaging data analysis

Data were analyzed using ImageJ using the JACoP plugin to determine Pearson’s correlation coefficients. Fusion events were detected as sudden increases in fluorescent intensity as shown in Fig. 1 i and Video 1. Signal duration (Fig. 3 d) was defined as the time from the start of the event until the fluorescent signal was indistinguishable against the PM fluorescence. Fusion activity was defined as the number of events over the course of a time-lapse experiment, which varied between experiments but was typically between 1 and 5 min. On average, ≥15 cells were imaged per condition in ≥5 different imaging windows. Shown are representative experiments replicated in independent experiments.

### Exosome isolation

Exosomes were prepared from the supernatant of 24-h–cultured HeLa cells as depicted in Fig. S1 c. In brief, exosomes were purified from the cultured media with exosome-free serum. Differential centrifugations at 500 *g* (2 × 10 min), 2,000 *g* (2 × 15 min), and 10,000 *g* (2 × 30 min) eliminated cellular debris, and centrifugation at 70,000 *g* (60 min) pelleted exosomes. The exosome pellet was washed once in a large volume of PBS, centrifuged at 70,000 *g* for 1 h, and resuspended in 200 µl PBS.

### RT-PCR

Total RNA was isolated with TRIzol reagent (Invitrogen) according to the manufacturers’ protocol. RNA was converted to cDNA using the AMV reverse transcription system (Promega). In brief, 1 mg RNA was incubated with 250 ng random primers for 5 min at 65°C followed by cDNA synthesis using 5 U AMV reverse transcription system for 45 min at 42°C in a total volume of 20 ml. Semiquantitative PCR reactions were performed with SYBR green I master using the LightCycler480 System (Roche Diagnostics) and consisted of 10 min incubation at 95°C followed by 45 cycles of 10 s at 95°C, 15 s at 60°C, and 15 s at 72°C. Amplification and melting curves were analyzed using the LightCycler480 Software release 1.5.0. The following primers were used for SNAP23 RT-PCR: forward, 5′-AACCCAGGATTCTCCTCGTA-3′, and reverse 5′-GTTGGGGTGTCCGAGTTG-3′; and for syntaxin-4 RT-PCR: forward, 5′-TTGATGAGCTCCACGAATTG-3′, and reverse, 5′-ATAGAGCCCCAGAAGGAGGA-3′.

### Statistical analysis

We performed statistical analysis (Student’s two-tailed *t* test for significance) using Prism (6.0; GraphPad Software). Unpaired analysis was used unless specified otherwise. Whiskers in Tukey’s box plots represent 1.5 times the interquartile distance or the highest or lowest point, whichever is shorter. Any data points beyond these whiskers are shown as dots. Data distribution was assumed to be normal, but this was not formally tested.

### Phosphoproteomics

Our titanium dioxide (TiOx)-based phosphoproteomics workflow was essentially the same as described by Piersma et al. (2015). In brief, cells were lysed in denaturing lysis buffer supplemented with phosphatase inhibitors, lysates were digested overnight with sequence grade–modified trypsin (Promega), and 0.5-mg aliquots were desalted on SepPak-C18 cartridges (Waters). Then, phosphopeptides were captured through metal oxide affinity chromatography with in-house–prepared TiOx STAGE tips (packed with 2.5 mg 10-µm TiO2 beads; GL Sciences) in the presence of 300 mg/ml lactic acid to suppress background binding of acidic peptides, eluted with piperidine, desalted with in-house–prepared STAGE tips packed with SDB-XC (3M Empore), dried, and redissolved, and then 50% was injected into a nano–liquid chromatography–tandem mass spectrometry system (Ultimate 3000 nanoLC coupled on-line to a QExactive mass spectrometer; Thermo Fisher Scientific) operated as described previously by Piersma et al. (2015). Protein identification was performed using MaxQuant software (1.5.2.8; Cox and Mann, 2008) with a FASTA file covering human canonical proteins and isoforms contained in the Swiss-Prot release of September 2015 (20,197 entries) using the following settings:

The enzyme specificity was set to trypsin with a maximum tolerance of two missed cleavages. Serine, threonine, and tyrosine phosphorylation (+79.966330 D), methionine oxidation (Met; +15.994915 D), and N-terminal acetylation (N-terminal; +42.010565 D) were treated as variable modifications, and cysteine carboxamidomethylation (Cys; +57.021464 D) was as fixed. Peptide precursor ions were searched with a maximum mass deviation of 4.5 ppm, and fragment ions were searched with a maximum mass deviation of 20 ppm. Peptide, protein, and site identifications were filtered at an 1% false discovery rate using the decoy database strategy. Seven amino acids was used for minimal peptide length, the minimum Andromeda score for modified peptides was set at 40, and the corresponding minimum delta score was set at 17. Proteins that could not be solely differentiated based on tandem mass spectrometry spectra were clustered as protein groups. Peptide identifications were propagated across samples using the match-between-runs option, and searches were performed using the label-free quantification option. This method was also described previously by Piersma et al. (2015).

### Label-free phosphopeptide quantification

Phosphopeptides were quantified by their extracted ion chromatograms, and for each sample, phosphopeptide intensities were normalized on the median intensity of all identified peptides in the sample. R was used for normalization and statistical testing. Fold change and p-values were calculated from replicates using a two-tailed Student’s *t* test. Phosphopeptides were considered significantly differential at P < 0.05, the match-between-runs option in MaxQuant was used, and missing values were excluded from subsequent statistical analysis. Before biological group comparisons, quantitative values from replicates were averaged. In each group, the *t* test required at least two quantitative values. P-values were not corrected for multiple hypothesis testing.

Cluster analysis of differential phosphopeptides was performed using hierarchical clustering in R. For each phosphopeptide, phosphopeptide intensities were normalized to zero mean and unit variance. Subsequently, for phosphopeptide clustering, the Euclidean distance measure was used, and for sample clustering metrics, the Ward linkage and the 1-Pearson correlation distance were used. This method was published by Piersma et al. (2015).

### Online supplemental material

Fig. S1 shows the characterization of the CD63-pHluorin reporter in HeLa and HUVEC cells. Fig. S2 shows that CD63–C-term–pHluorin and CD63-pHuji can be used to distinguish MVB fusion events from the fusion of acidic compartments without ILVs. Fig. S3 shows the protocol for isolating cell-associated EVs, the fusion signal duration of TSPAN-pHluorins in HEK293 cells, that tetherin is not responsible for prolonged fusion duration of CD63-pHluorin, and that the second messenger cAMP increases fusion activity. Fig. S4 shows a network analysis of differentially phosphorylated proteins and interactors upon histamine stimulation in HeLa and HUVEC cells and GO-term enrichment for this network. Furthermore, Fig. S4 shows the effect of SNAP23-CΔ9 on EV release and localization of SNAP23 S110A in HeLa cells. Videos 1, 2, 3, and 4 show time-lapse imaging of CD63-pHluorin in HeLa cells (Video 1), SiHa cells (Video 2), HUVEC cells (Video 3), and MSCs (Video 4). Video 5 shows dual-TIRF time-lapse imaging of CD63–C-term–pHluorin and CD63-pHuji in HeLa cells. Video 6 explains the CLEM-3D tomography procedure linking a CD63-pHluorin event during live microscopy to an MVB–PM fusion profile at EM resolution. Videos 7 and 8 show time-lapse imaging of NPY-pHluorin and VAMP2-pHluorin in HeLa cells, respectively. Video 9 shows 3D heatmap time-lapse imaging of a CD63-pHluorin fusion event in HeLa corresponding with Fig. 3 b. Videos 10 and 11 show time-lapse imaging of CD81-pHluorin and CD9-pHluorin, respectively, in HeLa cells. Table S1 shows histamine-induced phosphorylation sites of proteins of interest. Table S2 shows SNARE protein enrichment in exosomes versus cells of various B cell lines as in Baglio et al. (2016). Table S3 shows the complete dataset of the phosphoproteomic experiment.

## Supplementary Material

Supplemental Materials

Table S3 (Excel)

Video 1

Video 2

Video 3

Video 4

Video 5

Video 6

Video 7

Video 8

Video 9

Video 10

Video 11
