# Correction: Quantifying exosome secretion from single cells reveals a modulatory role for GPCR signaling

**DOI:** 10.1083/JCB.20170320601192018c

**Published:** 2018-03-05

**Authors:** Frederik Johannes Verweij, Maarten P. Bebelman, Connie R. Jimenez, Juan J. Garcia-Vallejo, Hans Janssen, Jacques Neefjes, Jaco C. Knol, Richard de Goeij-de Haas, Sander R. Piersma, S. Rubina Baglio, Matthijs Verhage, Jaap M. Middeldorp, Anoek Zomer, Jacco van Rheenen, Marc G. Coppolino, Ilse Hurbain, Graça Raposo, Martine J. Smit, Ruud F.G. Toonen, Guillaume van Niel, D. Michiel Pegtel

Vol. 217, No. 3, March 5, 2018. 10.1083/jcb.201703206.

The first version of this article published online included errors in Figure 4 that occurred during production. The graph in Fig. 4 H was inadvertently duplicated in Fig. 4 D.

**Figure 4.**
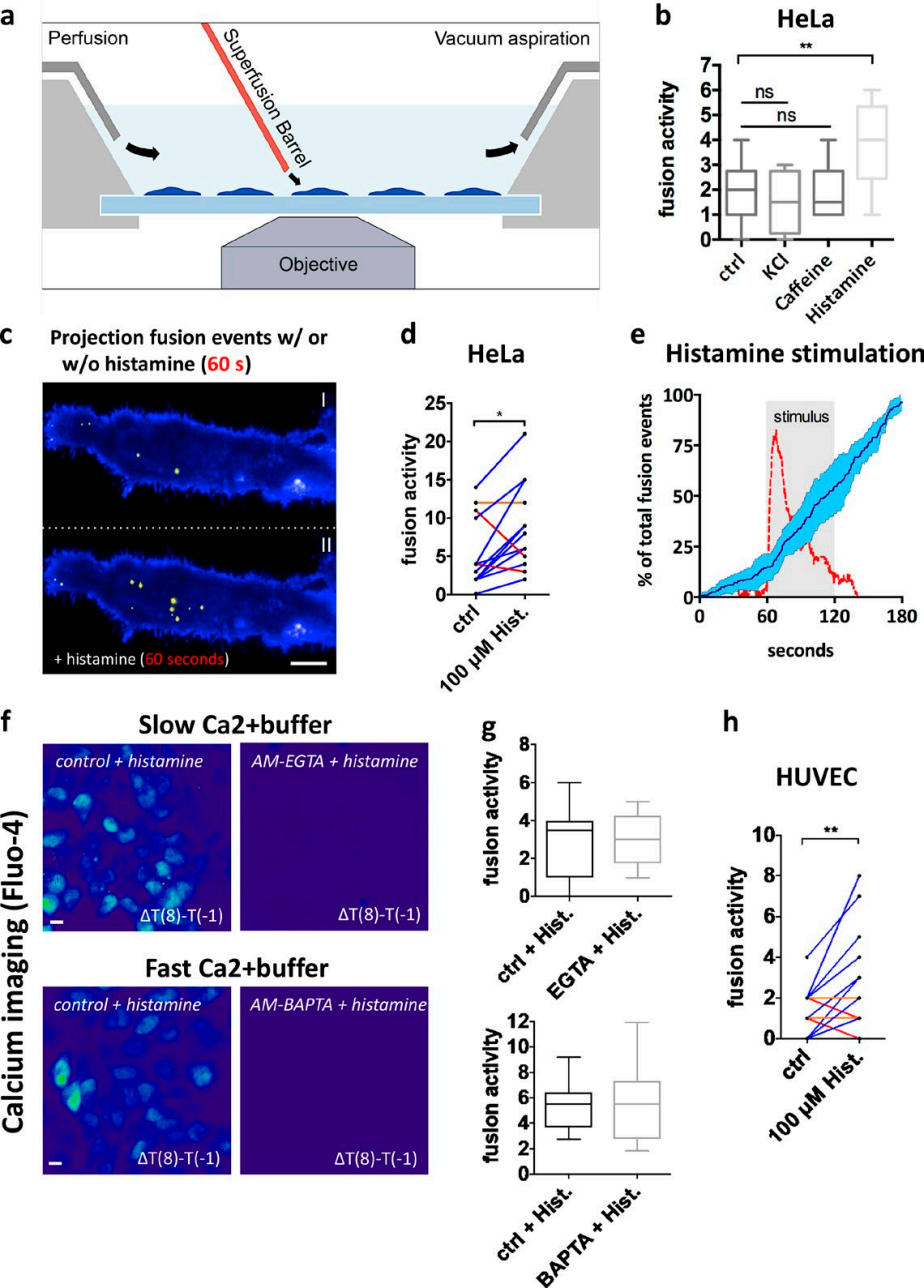
**GPCR activation triggers MVB–PM fusion in single cells in a calcium-independent manner. (a)** Schematic model of imaging setup. **(b)** Fusion activity of HeLa cells stimulated with KCl (70 mM), caffeine (20 mM), or histamine (100 µM). *n* ≥ 8 cells per condition. **(c)** Total projection of fusion events over a 60-s time course onto cells before (top) and after (bottom) stimulation with histamine (100 µM). Pseudocolored as in Fig. 1 j. **(d)** Measurement of individual HeLa cells (*n* = 14) before and during stimulation with histamine (100 µM). **(e)** Mean fusion kinetics of CD63-pHluorin HeLa cells (*n* = 6) showing the distribution of fusion events over time (dark blue line; SD is in light blue) and the calcium levels (red) during histamine stimulation (gray-shaded block). **(f)** Heat maps revealing calcium responses (measured by Fluo-4) upon histamine stimulation obtained by subtracting the fluorescent intensity values before stimulation from those after 8-s stimulation. Cells were nontreated or incubated with a buffer with fast (BAP​TA) or slow (EGTA) calcium-binding kinetics. Bars, 10 µm. **(g)** Quantification of fusion activity of histamine-stimulated HeLa cells in the presence of EGTA (top) or BAP​TA (bottom) buffers. *n* ≥ 10 cells per condition. **(h)** Measurement of individual HUV​EC cells (*n* = 30) before and after stimulation with histamine (100 µM). *, P < 0.05; **, P < 0.01 using Student’s two-tailed two-sample *t* test. All *t* tests were unpaired except for d and h. Whiskers in the box plots (b and g) represent 1.5 times the interquartile distance or the highest or lowest point, whichever is shorter.

Both the HTML and PDF versions of the article have been corrected. This error appears only in PDF versions downloaded on or before January 22, 2018. Rockefeller University Press apologizes for this regrettable error.

