# Supplementary material for: Quantifying exosome secretion from single cells reveals a modulatory role for GPCR signaling
Source: J Cell Biol. 2018 Mar 5;217(3):1129–42. doi: 10.1083/jcb.201703206 (PMC5839777; doi:10.1083/jcb.201703206)
Supplement: Supplemental Materials [file JCB_201703206_sm.pdf]

Verweij et al., https://doi.org/10.1083/jcb.201703206

JOURNAL OF CELL BIOLOGY

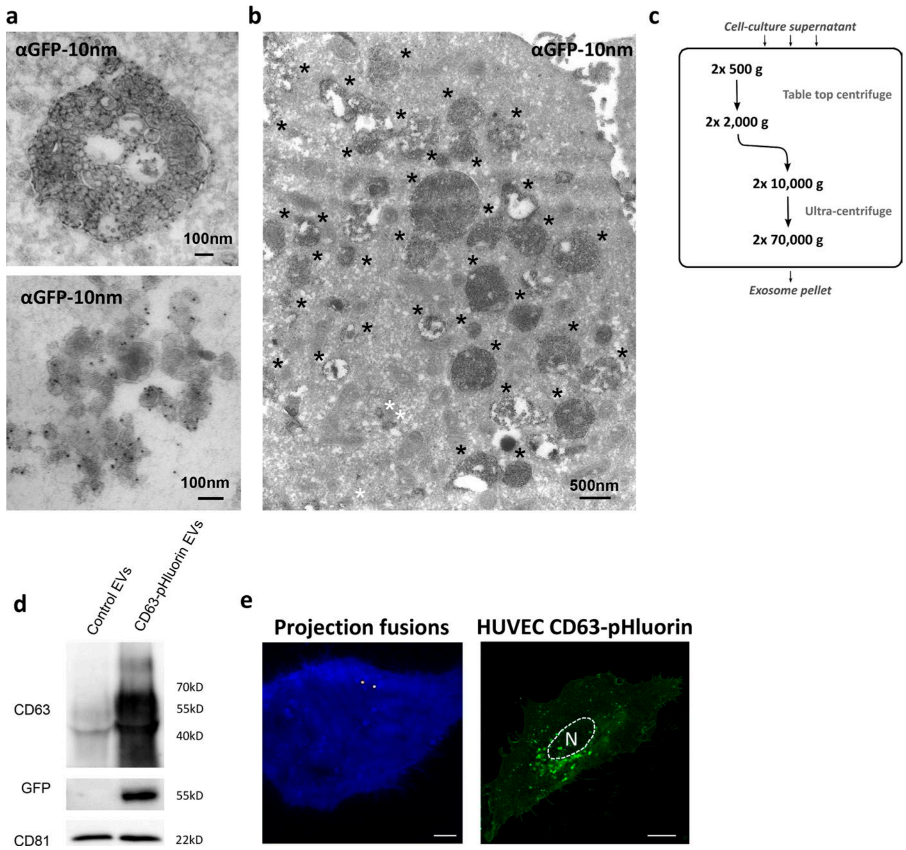

Figure S1. **Characterization of the CD63-pHluorin reporter in HeLa and HUVEC cells.** **(a)** EM pictures of an MVB of a CD63-pHluorin–expressing HeLa cell (top) and EVs purified by ultracentrifugation (bottom) labeled with gold particles (10 nm) coupled to anti-GFP antibody. **(b)** EM image showing numerous MVBs in the cytoplasm of a CD63-pHluorin–expressing HeLa cell. Asterisks indicate MVBs (black) or small endosomes (white). **(c)** Schematic overview of the exosome purification protocol by ultracentrifugation. **(d)** Western blot analysis of isolated EVs from CD63-pHluorin–transfected HeLa cells. **(e)** Left: total projection of fusion events (bright spots) over a time course of 1 min onto a HUVEC cell (blue). Right: representative example of a CD63-pHluorin–expressing HUVEC cell. N, nucleus. Bars, 10 µm.

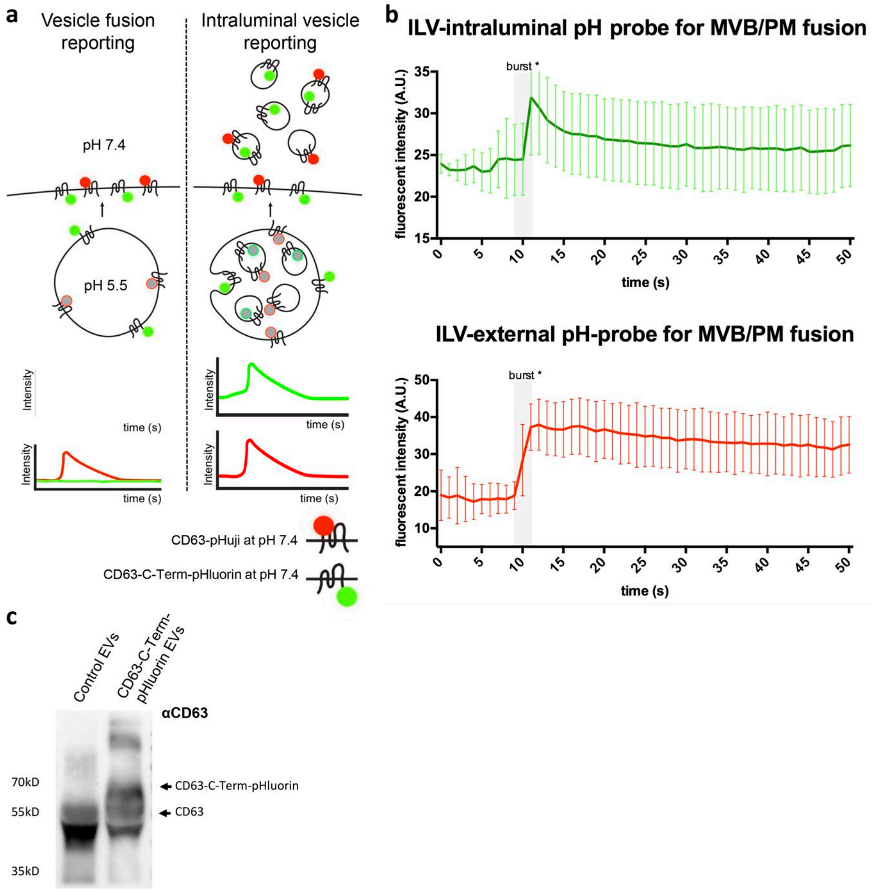

Figure S2. **CD63-pHluorin fusion events coincide with cargo externalization. (a)** To differentiate between the ILV signal (increase) and the signal coming from the LM, an alternative pHluorin-based reporter, CD63–C-term–pHluorin, was generated. With pHluorin placed at the C terminus instead of within EC1, CD63–C-term–pHluorin could not display (increased) fluorescence from the LM but only that of internal cargo exposed at the moment of fusion. Coexpression of CD63–C-term–pHluorin with a pH-sensitive red fluorescent version of CD63-pHluorin (CD63-pHuji) allowed for the differentiation between fusion of vesicles without (left) or with ILV cargo (right) based on the absence or presence of a fluorescent peak in the green channel at the moment of a burst in the red channel. **(b)** 12 events from three different cells were used to construct a mean fluorescent intensity profile synchronized at the moment of fluorescent increase in the red channel. The gray area marks the burst. Error bars indicate SD. **(c)** Western blot on EVs isolated from CD63–C-term–pHluorin HEK293 and control cells labeled for CD63.

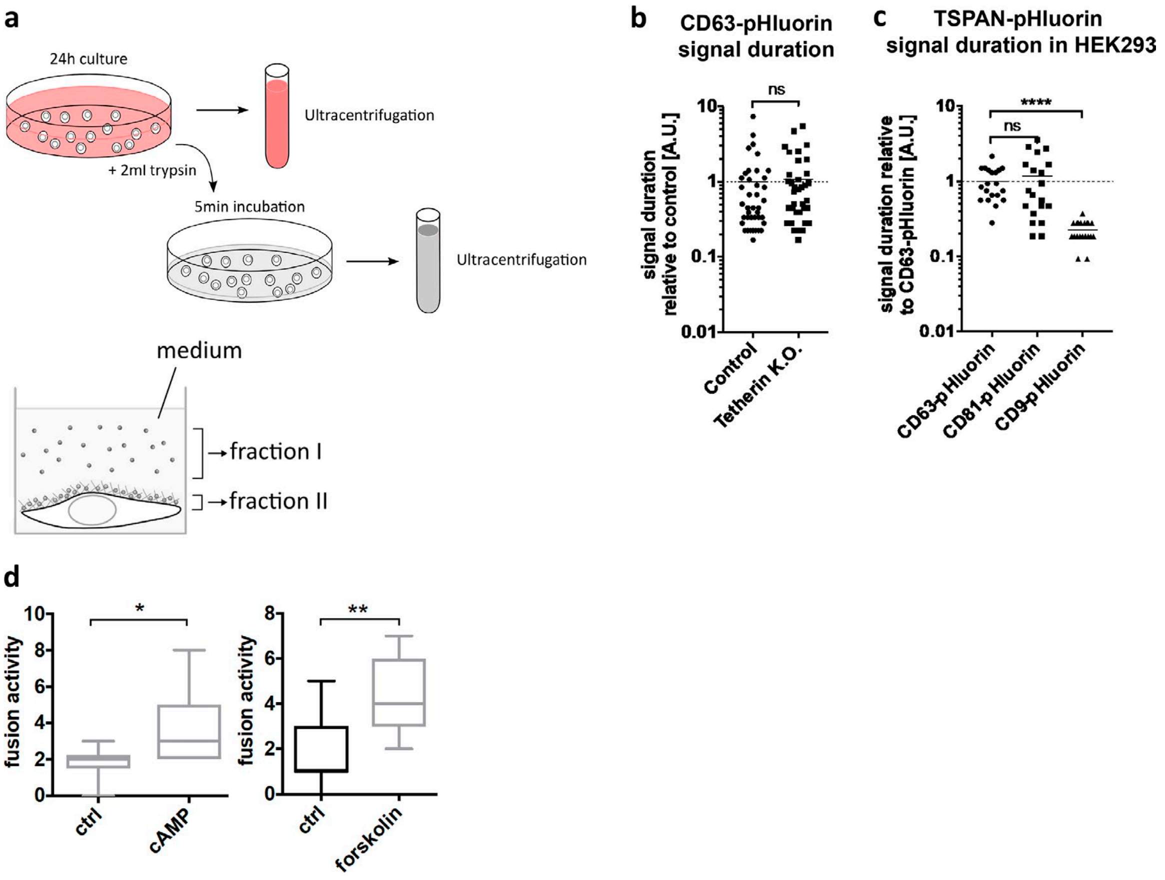

Figure S3. **Purification protocol of cell-associated EVs, tetherin is not responsible for prolonged fusion duration of CD63-pHluorin, fusion signal duration of TSPAN-pHluorins in HEK293 cells, and the second messenger cAMP increases fusion activity. (a)** Schematic representations of the procedure for exosome removal from the ECM using trypsin. In short, medium of 24-h cell culture was removed from the cells (fraction I). The remaining cells were trypsinized for 5 min. Exosomes from the trypsin fraction (fraction II) and fraction I were then isolated by differential ultracentrifugation. **(b)** Comparison between fusion durations of CD63-pHluorin in control and tetherin-knockout (KO) HeLa cells. n = 40 events per condition. **(c)** Direct comparison between fusion durations of CD81- and CD9-pHluorin relative to CD63-pHluorin as in Fig. 3 g but for HEK293 cells. n = 20 events per reporter. **(d)** Fusion activity of HeLa cells stimulated with a cell-permeable nonhydrolyzable cAMP derivate (8CPT-cAMP; 400 µM) and with forskolin (100 µM). $n \geq 6$ per condition. *, $P < 0.05$; **, $P < 0.01$; ****, $P < 0.0001$. Statistical analyses were performed using Student's two-tailed two-sample t test.

a

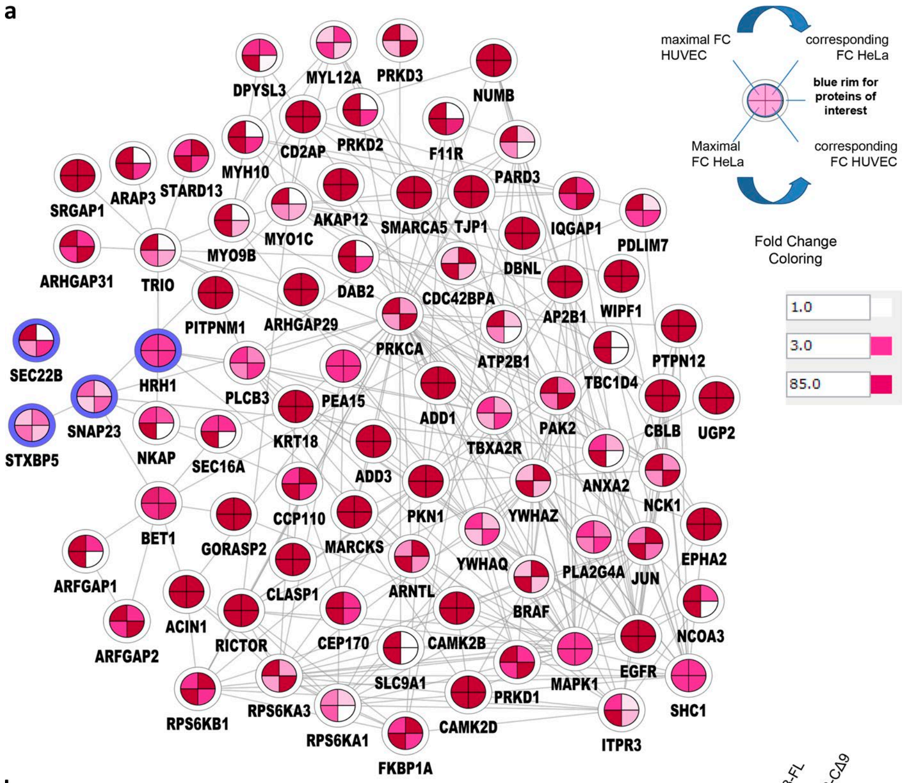

b

GO-ID	pathway description	count in gene set	false discovery rate
5886	plasma membrane	10	0,0272
16023	cytoplasmic membrane-bounded vesicle	6	0,0272
71944	cell periphery	10	0,0272
6906	vesicle fusion	3	0,0315
16050	vesicle organization	4	0,0315
1775	cell activation	5	0,0466
16192	vesicle-mediated transport	6	0,0475
51649	establishment of localization in cell	7	0,0475

c

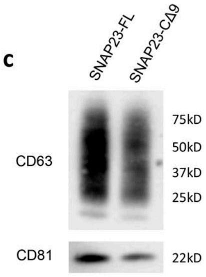

d

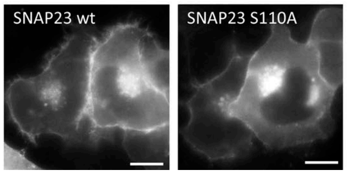

Figure S4. **Phosphoproteomic network analysis of histamine-stimulated cells, effect of SNAP23-CΔ9 on EV release, and localization of SNAP23 S110A in HeLa cells. (a)** Network of proteins of interest with altered phosphorylation levels upon histamine (100 µM) stimulation combined with differentially phosphorylated first (direct) and second (indirect) level interactors as identified by phosphoproteomics in HeLa and HUVEC cells. Proteins of interest are depicted with a blue rim. FC, fold change. **(b)** Table showing GO term enrichment for this protein network. **(c)** Western blot on EVs isolated from SNAP23-FL and SNAP23-CΔ9 HeLa cells labeled for CD63 and CD81. $n \geq 6$ per condition. **(d)** Comparison of SNAP23 localization in HeLa cells expressing GFP-SNAP23-WT and GFP-SNAP23-S110A. Bars, 10 µm.

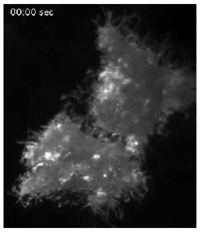

Video 1. **Time-lapse imaging of two CD63-pHluorin HeLa cells at 8× normal speed.** Shot at five frames per second.

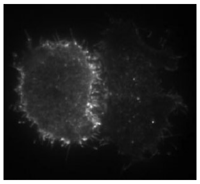

Video 2. **Time-lapse imaging of a CD63-pHluorin SiHa cell (cervix carcinoma) at 3× normal speed.** Shot at three frames per second.

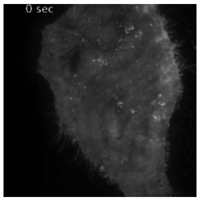

Video 3. **Time-lapse imaging of a CD63-pHluorin HUVEC cell at 4× normal speed.** Shot at two frames per second.

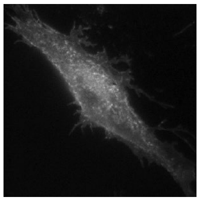

Video 4. **Time-lapse imaging of a CD63-pHluorin MSC cell at 1× normal speed.** Shot at 20 frames per second.

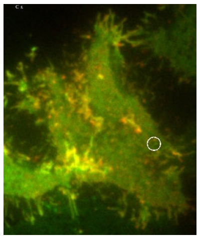

Video 5. **Dual-TIRF time-lapse imaging of CD63–C-term–pHluorin and CD63-pHuji HeLa cells at 5× normal speed.** White circles indicate fusion events of interest. Dual channel shot at one frame per second.

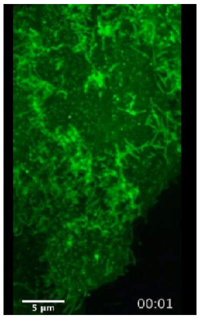

Video 6. **Video explaining the CLEM-3D tomography procedure linking a CD63-pHluorin event during live microscopy to an MVB–PM fusion profile at EM resolution.**

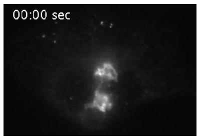

Video 7. **Time-lapse imaging of a NPY-pHluorin HeLa cell at 2× normal speed.** Shot at 20 frames per second.

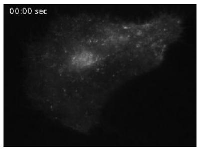

Video 8. **Time-lapse imaging of a VAMP2-pHluorin HeLa cell at 8× normal speed.** Shot at three frames per second.

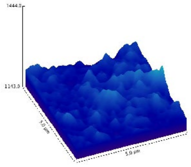

Video 9. **3D heatmap time-lapse imaging of a CD63-pHluorin fusion event in HeLa corresponding with Fig. 3 b.** Shot at 15 frames per second.

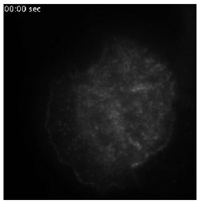

Video 10. **Time-lapse imaging of a CD81-pHluorin HeLa cell at 8× normal speed.** Shot at two frames per second.

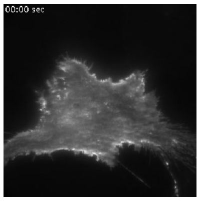

Video 11. **Time-lapse imaging of a CD9-pHluorin HeLa cell at 8× normal speed.** Shot at two frames per second.

Table S1. **Histamine-induced phosphorylation sites of proteins of interest**

Protein	Histamine-induced phosphorylation site
H1HR	Ser380
SNAP23	Ser110
STXBP5	Ser723/Ser759

Table S2. **Snare protein enrichment in exosomes versus cells of various B cell lines**

Protein name	FE[a] exo/cell
Syntaxin-binding protein 3	15.2
Isoform SNAP-23a of synaptosomal-associated protein 23	7.5
Isoform 1 of syntaxin-binding protein 6	4.6
Syntaxin-4	4.4
Syntaxin-12	4.0
Synaptogyrin-2	3.8
Vesicle-associated membrane protein 3	3.8
Syntaxin binding protein 2 isoform b	3.5
Isoform 1 of synaptophysin-like protein 1	3.4
Vesicle-associated membrane protein 5	3.0
Vesicle-associated membrane protein 2	3.0
Synaptic vesicle membrane protein VAT-1 homolog	2.2
Isoform 1 of vesicle-associated membrane protein 7	1.8
Isoform 1 of syntaxin-7	1.8
Isoform 1 of syntaxin-2	1.7
Isoform 1 of extended synaptotagmin-1	1.6
Syntaxin-11	1.6
Synaptobrevin homolog YKT6	1.5

[a]Fold enrichment.

Table S3 is a separate Excel file showing the complete dataset of the phosphoproteomic experiment.
